# Forest terpenes and stress: Examining the associations of filtered vs. non-filtered air in a real-life natural environment

**DOI:** 10.1016/j.envres.2025.121482

**Published:** 2025-03-25

**Authors:** Chaja M. Levy, Anne M. Riederer, Christopher D. Simpson, Amanda J. Gassett, Abigail J. Gilbert, Michael H. Paulsen, Lalith K. Silva, Deepak Bhandari, Cody A. Newman, Benjamin C. Blount, Peter H. Kahn, Gregory N. Bratman

**Affiliations:** a School of Environmental and Forest Sciences, University of Washington, USA; b Department of Environmental and Occupational Health Sciences, University of Washington, USA; c Division of Laboratory Sciences, National Center for Environmental Health, U.S. Centers for Disease Control and Prevention, USA; d Department of Psychology, University of Washington, USA

**Keywords:** Terpenes, Forest, Nature, Health, Stress

## Abstract

Human health may benefit from exposure to a class of biogenic volatile organic compounds (BVOCs) consisting of isoprene units, known as terpenes. In this double-blind, randomized crossover trial, participants sat in a forest for two 60-min sessions, one in which terpenes were filtered out of the ambient air they breathed, and another in which they were not, separated by a minimum of an eight-day washout period. The primary outcome was the high frequency (HF) component of heart rate variability (HRV; measured continuously). Secondary outcomes included skin conductance levels (SCL) (measured continuously), self-reported stress and affect (measured every 20 min), blood pressure, heart rate, cortisol and inflammatory cytokines (measured before and after sessions). Serum concentrations of terpenes (measured before and after sessions) were also assessed to investigate the association of absorbed dose with these outcomes. We did not observe a significant association of filter condition with most outcomes; although the trends for affect, systolic blood pressure, cortisol, TNF-α, and CRP were all in the hypothesized direction. We did observe a significant association with interleukin-6, which was −0.19 pg/mL lower in the terpenes-on vs. terpenes-off condition, adjusted for baseline (95 % CI: −0.35, −0.03); and SCL over the session as a whole. A sensitivity analysis of the subset of data from participants who completed both conditions supports these findings and revealed additional significant associations with SCL (95 % CI: −1.87, −0.05); and TNF-α (95 % CI: −2.63, −0.10). To our knowledge, this is the first RCT to filter terpenes from ambient air during forest contact.

## 1. Introduction

Nature contact has been linked to beneficial psychological and physiological outcomes in humans (Bratman et al., 2019). As outlined by Frumkin et al. (2017), these include positive associations with stress, anxiety, affect, depressive symptoms, prosocial behavior, and attention (Beil and Hanes, 2013; Berman et al., 2008; Bratman et al., 2015; Ewert and Chang, 2018; Hong et al., 2018; Hunter et al., 2019; Jiang et al., 2014; Kaźmierczak, 2013; Putra et al., 2020; Ward Thompson et al., 2016; Zhao et al., 2022). Additionally, nature contact has been associated with improved sleep, reduced mortality, and reduced risk of Type II diabetes (Astell-Burt et al., 2014; Astell-Burt and Feng, 2020; Bodicoat et al., 2014; Gascon et al., 2016; James et al., 2016; Johnson et al., 2018; Shin et al., 2020). However, further research is needed regarding causal mechanisms underlying these associations.

Shinrin-yoku, also known as “forest bathing,” is a specific set of human-nature interactions that has been shown in some studies to have beneficial associations with mood, anxiety, depressive symptoms, neuroimmunological, and physiological outcomes (Kobayashi et al., 2017, 2018; J. Lee et al., 2015; Li et al., 2007; Park et al., 2007; Park et al., 2000; Song et al., 2015, 2017; Tsunetsugu et al., 2013; Yu et al., 2017), though other studies have reported mixed results (see Horiuchi et al., 2014; Kavanaugh et al., 2022; Kim et al., 2022; Stigsdotter et al., 2017). This body of research posits that the effects of forest bathing may be due in part to anti-inflammatory, psychoneuroimmunological processes that are associated with exposures to a class of biogenic volatile organic compounds (BOVCs) that exist in forest air (Bratman et al., 2024; Cho et al., 2017; Hartig et al., 2014; Kuo, 2015; Li et al., 2008; Satyawan et al., 2022). BVOCs are volatile compounds that are emitted by plants and serve a variety of functions, including aiding plant reproduction and defending against herbivory (Antonelli et al., 2020; Dudareva et al., 2006). Shinrin-yoku focuses on the potential human health effects that come from exposures to a certain class of these BVOCs that are isoprenoids, known as terpenes.

In animal and *in vitro* experiments, terpenes have been shown to be associated with decreases in inflammation and anti-tumorigenic outcomes (Cho et al., 2017; De Almeida et al., 2017; Santos, 2004), including inhibited production of inflammatory cytokines, leukotrienes, and associated metabolites in human monocytes, such as TNF-α, IL-1β, Il-4, Il-5, IL-6, IL-8, and leukotriene B4 (Bastos et al., 2011; De Cássia Da Silveira E Sá et al., 2013; Hart et al., 2000; Juergens et al., 1998, 2004). Additionally, terpene or essential oil exposure has been associated with anti-inflammatory outcomes in asthma patients, reduced anxiety and depressive symptoms, reduced stress, increased positive affect, and improved cognitive function and sleep (Chen et al., 2022; Fung et al., 2021; Goes et al., 2012; Juergens et al., 2003; Kerr et al., 2021; Lee et al., 2017; Moss and Oliver, 2012; Woo et al., 2023). Potential effects of terpenes on psychophysiology, neurotransmitter production, and biomarkers may be mediated by signal transduction pathways (Cui et al., 2022; Faturi et al., 2010), and/or changes in the production of transcription factor proteins that regulate gene transcription (e.g., NF-κB) (D.-S. Kim et al., 2015; T. Kim et al., 2020; Rufino et al., 2014).

Our study was designed to assess the impacts of inhalation of terpenes in ambient forest air, much of which enters through the nose during respiration. Sense of smell, or olfaction, refers to the sensory process through which the olfactory system and brain interprets odors from the surrounding environment (Doty, 2001). Olfaction is linked to affective responses and effects on cognitive function through subjective experiences of odors (Bratman et al., 2024; Kontaris et al., 2020; Martin and Chaudry, 2014; Schiffman et al., 1995; Seubert et al., 2008). Additionally, effects may occur through sub-threshold pathways which bypass conscious cognition – including cases in which volatile compounds may travel via the olfactory or trigeminal nerves into the brain or enter systemic circulation and cross the blood-brain barrier, with subsequent effects on neurotransmission (Chioca et al., 2013; Fung et al., 2021; Hanson and Frey, 2008).

Here, we aimed to fill a critical gap in knowledge on the association of terpenes in ambient air with human well-being by examining whether inhalation of filtered vs. non-filtered air in a forest setting is associated with a set of psychophysiological and immunological outcomes, using a randomized double-blind crossover trial design. The primary outcome was the HF component of HRV, a measure of parasympathetic nervous system activity. Secondary outcomes included measures of skin conductance levels (SCL), blood pressure, and HR (i.e., measures of sympathetic nervous system activity, or correlates of stress), self-reported stress and affect, and levels of cortisol and inflammatory cytokines in serum. Terpene concentrations in serum were measured to assess the degree of association of absorbed dose with study outcomes. We hypothesized that inhalation of non-filtered forest air would be associated with greater increases in HF HRV and positive affect, and greater decreases in SCL, blood pressure, HR, self-reported stress, negative affect, and levels of cortisol and inflammatory cytokines in serum compared to the condition with filtered air. Additionally, we hypothesized that these outcomes would be associated with absorbed dose of forest terpenes.

To our knowledge, no study has been conducted that concurrently investigated real-world filtration vs. non-filtration of terpenes in ambient forest air, absorption of these terpenes as measured via serum levels, and short-term impacts of experimental vs. control conditions on psychophysiological and immunological outcomes in humans.

## 2. Materials and methods

### 2.1. Participants

We enrolled 43 adult participants between July 12, 2022, and September 21, 2023. Our target sample size of 40 participants was based on a power calculation to detect minimum differences in HF-HRV (details provided in Supplementary Materials). Participants were recruited from the Tacoma and Seattle, WA area using physical flyers placed in community centers, libraries, and local universities, and electronic listings on Craigslist and the University of Washington (UW) Institute of Translational Health Sciences study recruitment site. Potential participants were screened via phone call and excluded if they were pregnant, smoking, not fluent in English, and/or had a current or prior diagnosis of neurologic, hypertensive, psychiatric, or respiratory disorder, or anosmia/hyposmia. Participants were also excluded if they were prescribed a short list of prescription medications known to influence terpene metabolic pathways and/or short-term inflammatory biomarkers including beta-blockers, antibiotics, statins, hypertension medications, steroid medications, and diabetes control medications, or if they were unable to walk for 15–20 min on unsteady ground. Eligible participants were asked to avoid certain consumer products, foods, beverages, cleaning products, alcohol, marijuana, e-cigarettes, and supplements that contain terpenes in the 24 h leading up to their forest-sitting experience (see Supplementary Materials for a complete list).

The clinically validated University of Pennsylvania Smell Identification Test (UPSIT; Sensonics International, Haddon Heights, NJ) was administered to determine whether participants had anosmia/hyposmia (full or partial smell loss; Doty et al., 1989). The UPSIT is a 40-item, self-administered “scratch-and-sniff” test that uses microencapsulated odorants that are released by scratching designated spaces on a paper test booklet. Summed scores were used to evaluate olfactory function and exclude participants with undiagnosed full or partial smell loss (summed UPSIT score ≤18; Doty, n.d.).

The study protocol was approved by the UW Institutional Review Board (approval number STUDY00013134). This trial was registered at www.clinicaltrials.gov (ClinicalTrials.gov identifier: NCT05316597) on March 9, 2022. Division of Laboratory Sciences analysis of deidentified specimens was reviewed by CDC and deemed not engaged in human subject research and was conducted consistent with applicable law and CDC policy. All participants provided written informed consent. Participants received $180 in the form of a cash card or gift card upon study completion ($90 per session). A $60 bonus for completing the second session was introduced with UW IRB approval in 2023 to reduce attrition.

### 2.2. Study design

Following enrollment, participants were randomly assigned to a sequence of two sessions using a computer-randomized order created by an investigator who was not a member of the field team. To control participants’ inhalation of terpenes in the forest air, participants wore a powered air purifying respirator (PAPR; 3M^™^ Versaflo^™^ Powered Air Purifying Respirator, TR-800-PSK/94248; 3M, St. Paul, MN). In one session, the PAPR mask was fitted with a particle-only filter (3M^™^ Versaflo^™^ High-Efficiency Filter, TR-6710N-5) to allow for the inhalation of forest terpenes (terpenes-on). In the other session, the mask was fitted with a charcoal filter (3M^™^ Versaflo^™^ Organic Vapor/HEPA Cartridge, TR-6510N) that removed particles, terpenes, and other BVOCs from the breathing zone (terpenes-off). Equipment labels were covered, and filters were labeled “A” (later revealed to be terpenes-on) or “B” (later revealed to be terpenes-off) to blind the study field team and participants to treatment assignment. A washout period of at least eight days between sessions was included to reduce carryover effects (Sibbald and Roberts, 1998).

Forest-sitting sessions took place at the University of Washington Charles L. Pack Experimental Forest located near Eatonville, Washington during June to September in both 2022 and 2023 to avoid rain, snow, and cold weather. The sessions were 60 min each and took place between 10:00 a.m. and 4:00 p.m. in a stand of old- and second-growth Douglas Fir, Western Red Cedar, and other conifers accessible by a 10-min drive on an unpaved road from the Pack Forest Conference Center. Participants were seated in a comfortable chair, but the session was otherwise unscripted.

### 2.3. Measures

The high frequency (HF) component of heart rate variability and skin conductance levels were measured continuously. Blood pressure and heart rate assessments, as well as serum samples were gathered before and after the 60-min forest-sitting sessions, and self-reported stress and affect were measured at the beginning and at 20 min-intervals of the 60-min sessions.

#### 2.3.1. High-frequency heart rate variability

High Frequency (HF) HRV was measured in milliseconds squared (ms^2^) using a portable, continuous electrocardiogram sensor (Ecg-Move4; Movisens^®^, Karlsruhe, Germany) worn directly on the chest with adhesive electrodes. We applied a natural logarithmic transformation to 5-min averages of HF HRV (ln-HF HRV) following Shaffer and Ginsberg (2017) and then winsorized the averages.

#### 2.3.2. Blood pressure and heart rate

Systolic and diastolic blood pressure were measured with an automated cuff monitor (GE Healthcare CARESCAPE V100 Vital Signs Monitor; GE Healthcare, Chicago, IL) in millimeters of mercury (mmHg). HR was also measured with the automated cuff monitor in beats per minute (BPM).

#### 2.3.3. Skin conductance levels

SCLs were measured in microsiemens (μS) using a portable, continuous electrodermal sensor (EdaMove4; Movisens^®^, Karlsruhe, Germany) worn on a wristband and attached to the palm with adhesive electrodes.

#### 2.3.4. Affect

General levels of positive and negative affect were assessed using the 20-item Positive and Negative Affect Schedule (PANAS; Watson et al., 1988) during baseline measurements and state levels were assessed during the forest-sitting sessions using the 10-item, shortened PANAS (I-PANAS-SF; Thompson, 2007). The PANAS is designed to assess affect over the past month and includes 10 items on positive affect (e.g., “Interested,” “Active”) and 10 on negative affect (“Distressed,” “Irritable”). Each item is rated on a 5-point scale, ranging from 1 (“very slightly or not at all”) to 5 (“extremely”) with sum positive and negative affect scores calculated. In the current study, the PANAS showed good reliability for positive affect (Cronbach’s α = 0.88) and negative affect (Cronbach’s α = 0.85). General levels of affect are included in Supplemental Materials. The state-level, 10-item shortened PANAS was the measure used to assess change during the forest-sitting sessions. On the I-PANAS-SF, 5 items assess positive affect (e.g., “Inspired”, “Alert”), and five assess negative affect (“Upset”, “Hostile”) at the present moment. Each item is rated on a five-point scale, ranging from 1 (“very slightly or not at all”) to 5 (“extremely”) from which sum positive and negative affect scores were calculated. In the current study, I-PANAS-SF showed good reliability for positive affect (Cronbach’s α = 0.86) and lower reliability for negative affect (Cronbach’s α = 0.40).

#### 2.3.5. Self-reported stress

General levels of perceived stress were assessed using the four-item version of the Perceived Stress Scale (PSS-4) designed to assess perceptions of stress over the last month (e.g., “In the last month, how often have you felt that you were unable to control the important things in your life?”; Cohen et al., 1983). Each item was rated on a 5-point scale, ranging from 0 (“never”) to 4 (“very often”) and a sum score was calculated. In the current study, PSS-4 showed good reliability (Cronbach’s α = 0.78).

A state-level, 1-item state-level stress assessment was used to assess change during the forest-sitting sessions. Participants were asked to rate to what extent they felt stressed at the moment on a 5-point scale, ranging from 1 (“very slightly or not at all”) to 5 (“extremely”).

#### 2.3.6. Perception of forest setting

Different aspects of the subjective experience of the forest setting were assessed following each session using a combination of questions about the sensory experience and ratings of the perceived restorativeness of the forest. Perceived restorativeness was assessed using the adapted 11-item Perceived Restorativeness Scale (PRS-11) which asks participants to rate the degree to which they agreed with statements such as “Places like that are fascinating” using an 11-point scale, ranging from 0 (“not at all”) to 10 (“completely”; Pasini et al., 2014), Additionally, participants were asked to rate the pleasantness of their sensory experience of the forest for sight, smell, and visual experience on a 10-point scale ranging from 1 (“not pleasant”) to 10 (“extremely pleasant”). Participants were also provided an open-ended text box to describe further thoughts or feelings they had about the sensory experience of the forest. Smell perception data are reported in Supplementary Materials.

#### 2.3.7. Serum collection and handling

A trained phlebotomist or registered nurse collected approximately 5 mL of blood via venipuncture before and after each session using BD Vacutainer^™^ Push Button Blood Collection Sets into 6 mL BD Vacutainer^™^ Venous Blood Collection Tubes (BD, Franklin Lakes, NJ). On the day of collection, samples were stored at room temperature for 30 min to allow clotting, transferred to a cooler at 4 °C for approx. 1–4 h, then centrifuged at 3000 rpm for 10 min. After centrifugation serum aliquots were separated into 2 mL Fisherbrand^™^ Externally and Internally Threaded Cryogenic Storage Vials (Thermo Fisher Scientific, Waltham, MA) and stored at −80 °C before being transported on dry ice to the Centers for Disease Control and Prevention (CDC) Tobacco and Volatiles Branch (Atlanta, GA) and the UW Center for Studies in Demography and Ecology Biodemography Laboratory (Seattle, WA) for analysis.

#### 2.3.8. Serum cortisol and inflammatory biomarkers

Acute cytokine response was assessed using a custom three-plex enzyme immunoassay microarray (Quansys Biosciences, Logan, UT, part number 107749 GR) to measure serum levels of CRP, TNF-α, IL-6, and cortisol. Chemiluminescence was quantified using a Quansys Q-view Imager LS. An 8-point, 5-parameter standard curve was used to estimate cytokine concentrations and assay limits of detection (Q-view software, Quansys Biosciences, Logan, UT).

A competitive microplate enzyme immunoassay, previously validated for use with plasma (Munro and Stabenfeldt, 1984), was adapted to measure cortisol in serum extracts using a purified polyclonal anti-cortisol antibody and a method that has been previously described, R4866 (provided by C. Munro, UC Davis), and cortisol reference calibrators (Steraloids, catalog number Q3880; Doyle et al., 2019; Heller et al., 2018; Konishi et al., 2012; Trumble et al., 2010). For both acute cytokine and cortisol response analyses, all samples, standards, and controls were assayed in duplicate wells.

#### 2.3.9. Serum terpenes analyses

Concentrations of α-pinene, β-caryophyllene, β-myrcene, β-pinene, Δ-3-carene, and limonene were measured in serum using stable isotope dilution with headspace solid-phase microextraction (SPME) and gas chromatography-tandem mass spectrometry (HS-SPME GC-MS/MS) following Silva et al. (2020). Briefly, samples were thawed, mixed using a hematology mixer (Fisher Scientific Inc., Pittsburgh, PA), and a 0.50 mL aliquot dispensed into a 10 mL SPME vial. Purge-and-trap grade methanol was used to dilute pure chemicals into the primary standard stock solutions, which were used to prepare ten calibration solutions. In addition, pure isotopically labeled analogues were diluted into internal standard (ISTD) stock solutions. A 40 μL spike of the ISTD solution was added to each sample, which were then crimp-sealed and mixed with a vortexer (S/P Multi-Tube Vortexer, Baxter Diagnostics Inc., Minster, OH) for 2 min. Vials were placed on a Peltier-cooled sample tray (15 °C) on an Agilent 7890 GC (Agilent, Santa Clara, CA) coupled to an Agilent 7010 triple-quadrupole MS (Agilent, Santa Clara, CA) with a mounted CombiPAL autosampler and extra PAL autosampler arm (CTC Analytics AG, Zwingen, Switzerland). Samples were analyzed in positive ion electron-impact ionization with multiple reaction monitoring modes.

Blank and quality control samples were also prepared with a 40 μL ISTD spike. Laboratory blanks consisted of low-VOC water in sealed ampoules opened on the day of analysis. We also collected 7 field blanks on random days comprised of low-VOC water drawn through serum tubes and handled identically to the serum samples. Analyte-specific limits of detection (LOD) were calculated using 60 runs containing water blanks and four serum samples with different analyte concentrations (CLSI, 2012). The serum samples consisted of a serum blank and three low-concentration analyte-fortified samples prepared from a human-serum sample and used in the LOD evaluation (Silva et al., 2020). Resulting LODs were calculated at 0.050 μg/L, 0.056 μg/L, 0.085 μg/L, 0.032 μg/L, 0.162 μg/L, and 0.600 μg/L for α-pinene, β-pinene, β-myrcene, Δ-3-carene, limonene, and β-caryophyllene respectively.

#### 2.3.10. Terpene concentrations in forest air

Terpenes were measured in air at the forest site while participants were present using a method adapted from NIOSH Method 1552 (National Institute for Occupational Safety and Health, 1996) and detailed elsewhere, along with associations of these ambient air levels of terpenes with those detected in serum (Riederer et al. *in preparation*). Briefly, air was drawn using a GilAir^®^ Plus low-flow pump (Sensidyne^®^, St. Petersburg, FL, USA) via polypropylene tubing into a MilliporeSigma™Supelco^™^ CT420 proprietary glass thermal desorption tube optimized for terpene collection (MilliporeSigma, Burlington, MA, USA). The tube was mounted on a metal stand, near the participant, at PAPR filter height (~0.5 m above the ground). One field blank, consisting of a tube taken to the site and end caps momentarily opened but no air drawn through was collected for each day of sampling, while field duplicates were collected on alternating weeks. Primary samples, blanks and duplicates were analyzed for 22 terpenes using thermal desorption-gas chromatography–mass spectrometry (TD-GC/MS) at Eurofins Laboratory (Edmonton, Alberta, Canada). LODs varied by analyte and field sampling period with the lowest detectable mass 0.25 ng (Riederer et al. *in preparation*).

#### 2.3.11. Nature contact duration, frequency, and relatedness

Average nature contact duration, nature contact frequency, and nature relatedness were assessed at baseline. Questionnaire information is provided in the Supplementary Materials.

### 2.4. Procedures

Participants met research staff at a study site in Tacoma for informed consent, initial surveys, and measurements. Participants were then transported to Pack Forest (~1 h drive) in a vehicle fitted with a fan that blew air filtered through a charcoal filter (Vivosun, Ontario, CA) into their seating area to substantially reduce terpene exposure prior to their session. Upon arrival, they were outfitted with a PAPR with charcoal filter (zero filter) to prevent terpene inhalation during initial study protocols.

**Fig. 1.**
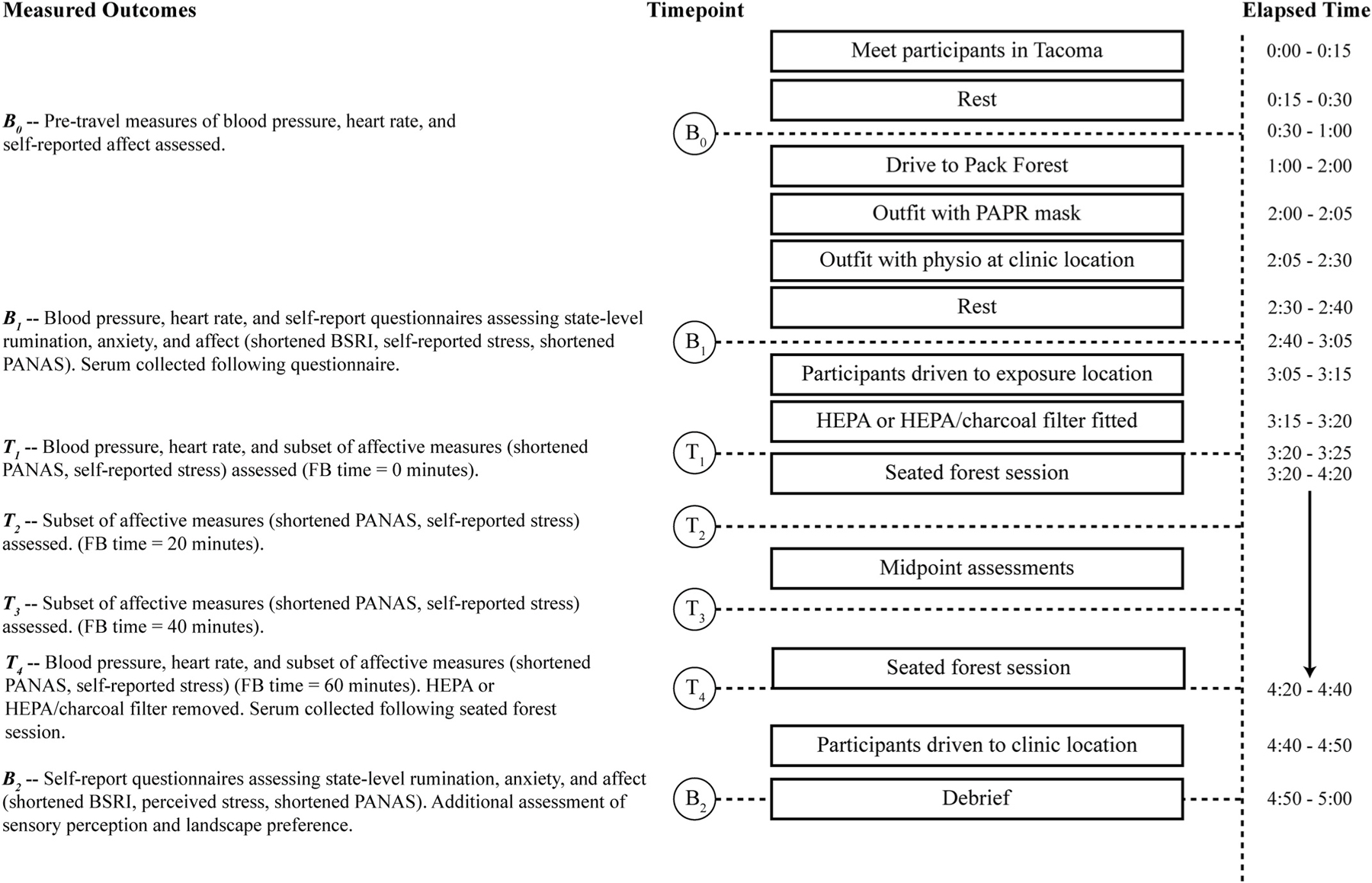
Overview of procedures for a study session.

Participants were then outfitted with the physiology equipment in a clinic room, rested for 10 min, and completed a survey with affective assessments and the baseline blood draw before being transported to the forest site where the zero filter was changed to the randomly assigned filter. Blood pressure, HR, and self-reported affect and stress were assessed at the beginning (T1; 0–5 min) and end of the session (T4; 55–60 min). Self-reported affect and stress were also assessed at two additional points: T2 (17.5–22.5 min) and T3 (37.5–42.5 min). A second blood sample was collected at T4. HF HRV and SCL were assessed continuously. A full daily protocol is outlined in Fig. 1.

### 2.5. Statistical methods

#### 2.5.1. Missing data

For 5 participants missing baseline ln-HF HRV, 5 min periods of B0 ln-HF HRV were used to predict the missing baseline measurements (see Supplementary Materials for details).

The I-PANAS-SF positive affect and negative affect outcomes are scored by summing the individual items. 16 (3.9 %) of the 414 questionnaires collected across all time points during participant-sessions were missing one or two items at one of the time points, although the data were typically complete for other time points. Because answers for the same item were modestly correlated between time points from the same day (mean R^2^ = 0.58) and performed better than other imputation approaches (random positive affect and negative affect item from the day, mean positive or negative affect score, and regression imputation), we used the mean available item value for missing observations. This methodology is similar to Shrive et al. (2006) but uses individual item row mean imputation, appropriate for longitudinal data (Engels and Diehr, 2003).

Machine values were used for the 35 % of left-censored IL-6 observations in the primary analyses. Sensitivity analyses were conducted substituting $\frac{limit\;\;of\;\;detection\;\;\left(LOD\right)}{\surd 2}$ and zero for the left-censored IL-6 observations. Participants who completed only one session were included in the analyses. We also conducted sensitivity analyses for the subset of cases in which participants completed both sessions and the complete data for the respective outcome was successfully assessed.

#### 2.5.2. Association of terpenes-on vs. terpenes-off filter with outcomes at T2 and T4 time points

Analyses were conducted in R (version 4.3.1; R Core Team, 2023) using the lme4 and lmerTest R packages (Bates et al., 2015; Kuznetsova et al., 2017). The criterion for statistical significance was *p* < 0.05.

We used mixed-effects regression and Equation (1) to evaluate the association of the terpenes-on vs. terpenes-off filter with T2 values of ln-HF HRV, SCL, self-reported stress, and self-reported positive and negative affect (I-PANAS-SF), and T4 values of systolic blood pressure, diastolic blood pressure, HR, inflammatory biomarkers, and cortisol, by session, controlling for baseline:

(1) $$Y_{is}=\beta_{0}+\beta_{1}baseline_{is}+\beta_{2}exposuregroup_{is}+\alpha_{i}+\varepsilon_{is}$$

where $Y_{is}$ is the observed mean outcome for participant i at T2 or T4, and session s where s is session 1 or session 2, $\beta_{0}$ is the mean of the $Y_{is}$ for the study population at T2 or T4 when they were assigned the terpenes-off group, $\beta_{1}$ is the estimated difference in $Y_{is}$ at T2 or T4 between two groups differing in baseline by 1 unit, $\beta_{2}$ is our primary coefficient of interest and is the estimated difference in $Y_{is}$ between the terpenes-off group and the terpenes-on group at T2 or T4, $\alpha_{i}$ is the random intercept for participant to account for repeated measures within participants, and $\varepsilon_{is}$ is the observation-specific error.

#### 2.5.3. Association of forest terpenes-on vs. terpenes-off filter with the pattern of outcomes across entire duration of session

We performed a two-way analysis of variance (ANOVA) to test the null hypothesis that there is no difference in the expected difference of ln-HF HRV, SCL, positive affect, negative affect, and self-reported stress at T1, T2, T3, and T4 between groups who differed by filter assignment controlling for baseline (see Supplementary Materials). The ANOVA is used to determine whether, taken as a whole, there are differences in the response pattern between the two conditions.

#### 2.5.4. Exploratory analyses

We calculated terpene absorbed dose as T4 minus baseline serum concentration for each individual analyte and a sum of all analytes. We examined associations between absorbed dose and the primary and other psychophysiological and immunological outcomes by replacing filter group with absorbed dose in Equation (1) in the Supplementary Materials. Additionally, we examined the association between absorbed dose and ln-HF HRV, skin conductance levels, self-reported stress, positive and negative affect levels across timepoints while controlling for baseline outcome and serum terpene concentrations (see Supplementary Materials).

Maximum likelihood estimation (MLE) was used to impute < LOD observations for serum α-pinene, β-myrcene, and Δ-3-carene (Finkelstein and Verma, 2001; Hewett and Ganser, 2007; Shoari and Dubé, 2018). Due to a low proportion of non-detects, $\frac{LOD}{\surd 2}$ was used to impute < LOD limonene concentrations. Σabsorbed dose was calculated as the sum of α-pinene, β-myrcene, Δ-3-carene, and limonene absorbed doses. Due to high (i.e., >50 %) proportions of non-detects, β-pinene and β-caryophyllene were excluded from the analyses.

## 3. Results

**Fig. 2.**
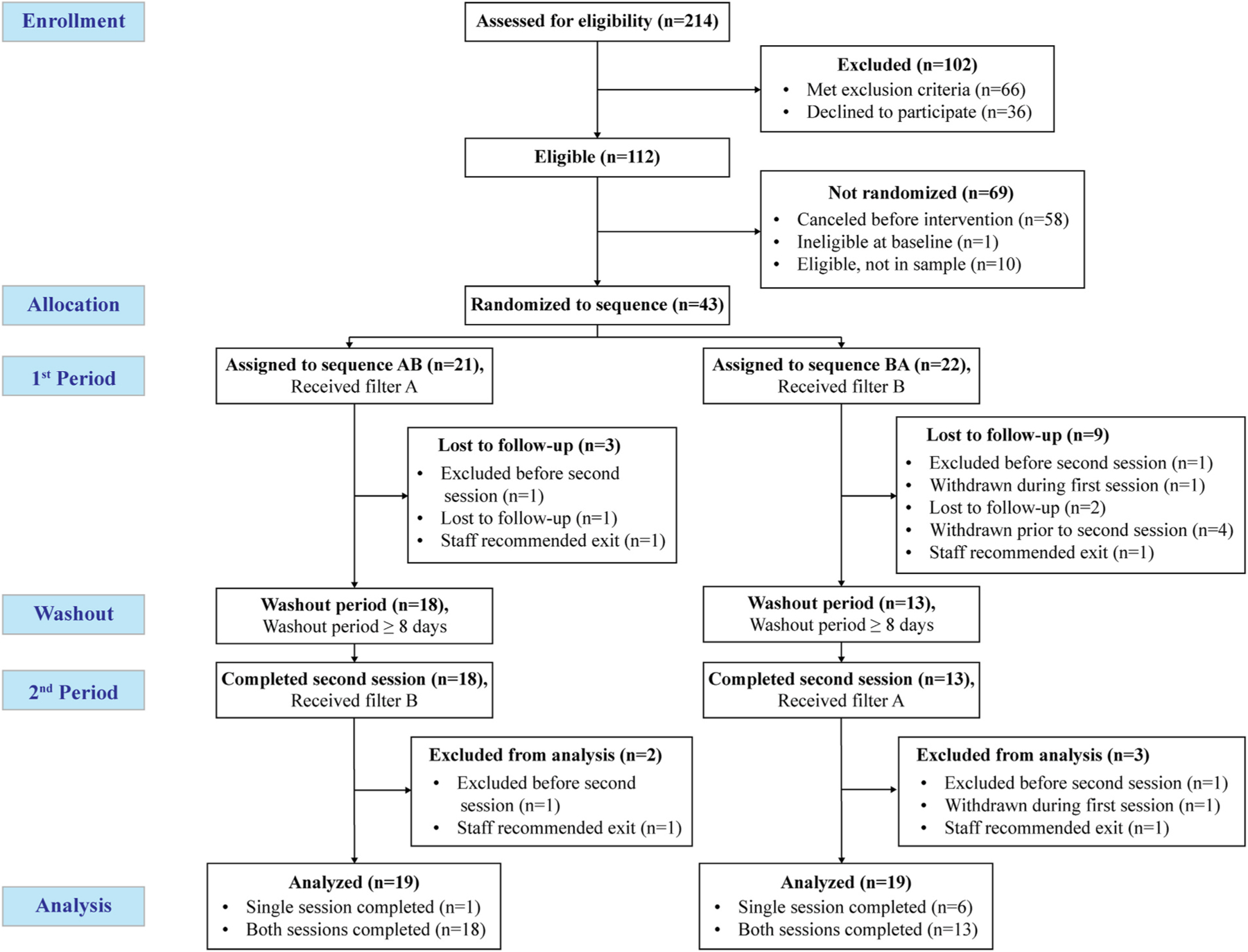
Enrollment, randomization, and retention of study participants among 43 adult participants in a randomized crossover terpenes-on vs. terpenes-off seated forest intervention trial conducted from June–September in both 2022 and 2023. *Note: A/B, terpenes-on/terpenes-off; B/A, terpenes-off/terpenes-on.*

A participant flow diagram is shown in Fig. 2. Twenty-one participants were assigned to sequence AB (terpenes-on followed by terpenes-off) and twenty-two were assigned to BA (terpenes-off followed by terpenes-on). Among AB sequence participants, two did not complete their second session due to moderate discomfort during the protocol or a shift in their self-reported answers to exclusionary criteria, and another was lost to follow-up. Among BA sequence participants, two did not complete their second session due to moderate discomfort during the protocol or a shift in their self-reported answers to exclusionary criteria, one withdrew during their first session, two were lost to follow-up, and four withdrew before their second session. The higher instances of withdrawal and loss to follow-up in the BA arm were likely due to a greater number of BA participants being enrolled during the first summer, when wildfire smoke and high heat events had a greater impact on data collection. This was due to considerations of safety and comfort of participants and study staff, and some sessions that therefore had to be canceled or rescheduled out of an abundance of caution. Wildfire smoke released from conifers also has a high concentration of α-pinene and β-pinene (Simpson et al., 2011), though we did not explicitly measure these potential effects.

Participant characteristics are shown in Table 1. The mean age was 35.1 ± 14.3 years and 51.2 % described their gender as female, 39.5 % described their gender as male, and 9.4 % described their gender as non-binary or preferred to self-describe. 2.3 % of participants described their race as American Indian or Alaska Native, 11.6 % as Asian, 7.0 % as Black or African American, 72.1 % as White, and 7.0 % as Other. Over half of participants had at least a bachelor’s degree and 58.3 % reported an annual household income below $80,000, approximately equal to Tacoma’s 2022 median annual household income of $80,784 (U.S. Census Bureau, 2022). Nearly half of participants were working (48.8 %), 18.6 % were students, and 32.2 % were not working or preferred not to answer.

Quality control experiments consisted of collecting air samples inside a PAPR mask fitted with the terpenes-on filter, and simultaneously collecting air samples from a collocated PAPR mask fitted with the terpenes-off filter, as well as samples from ambient air with no filters. The PAPR with the terpenes-off filter reduced terpene concentrations by >~70 % relative to the PAPR with the terpenes-on filter. However, contrary to expectations terpene concentrations in the co-located ambient samples were consistently lower (by an average of 4-fold) than the PAPR with the terpenes-on filter. Ambient samples were on average 50 % higher than the PAPR with the terpenes-off filter, but in some cases the ambient samples were lower than the collocated PAPR with the terpenes-off filter.

One possible explanation for this unanticipated result is that the PAPRs became contaminated with low levels of terpenes, including terpene-containing particles that collected on the PAPR filters during the course of the study. This potential contamination would likely not have impacted our heath analyses that compared the terpenes-on condition to the terpenes-off condition, since the PAPR with the terpenes-off filter reduced terpene concentrations by >~70 % relative to the PAPR with the terpenes-on filter and the in-vehicle filtration system reduced terpene concentrations by ~ >90 % (see Supplementary Materials). In testing of the filters after study completion, we were able to detect terpenes in the headspace above samples from the terpenes-on filter but not from the terpenes-off filter, suggesting that terpenes-on group was exposed to terpenes, while terpenes-off group would not have had such exposure from the filters.

### 3.1. Association of terpenes-on vs. terpenes-off filter with outcomes at T2 and T4

Counter to our hypothesis, we did not observe a significant association of the terpenes-on vs. terpenes-off filter with higher T2 values of ln-HF HRV and positive affect, or a significant association of the terpenes-on vs. terpenes-off filter with lower T2 values of negative affect or self-reported stress and T4 values of cortisol, TNF-α, or CRP. Although not statistically significant, the associations of the terpenes-on vs. terpenes-off filter with positive affect, negative affect, systolic blood pressure, cortisol, TNF-α, and CRP were all in the hypothesized direction (see Table 2). Figures displaying outcome trends over time by filter condition are presented in Supplementary Materials.

**Fig. 3.**
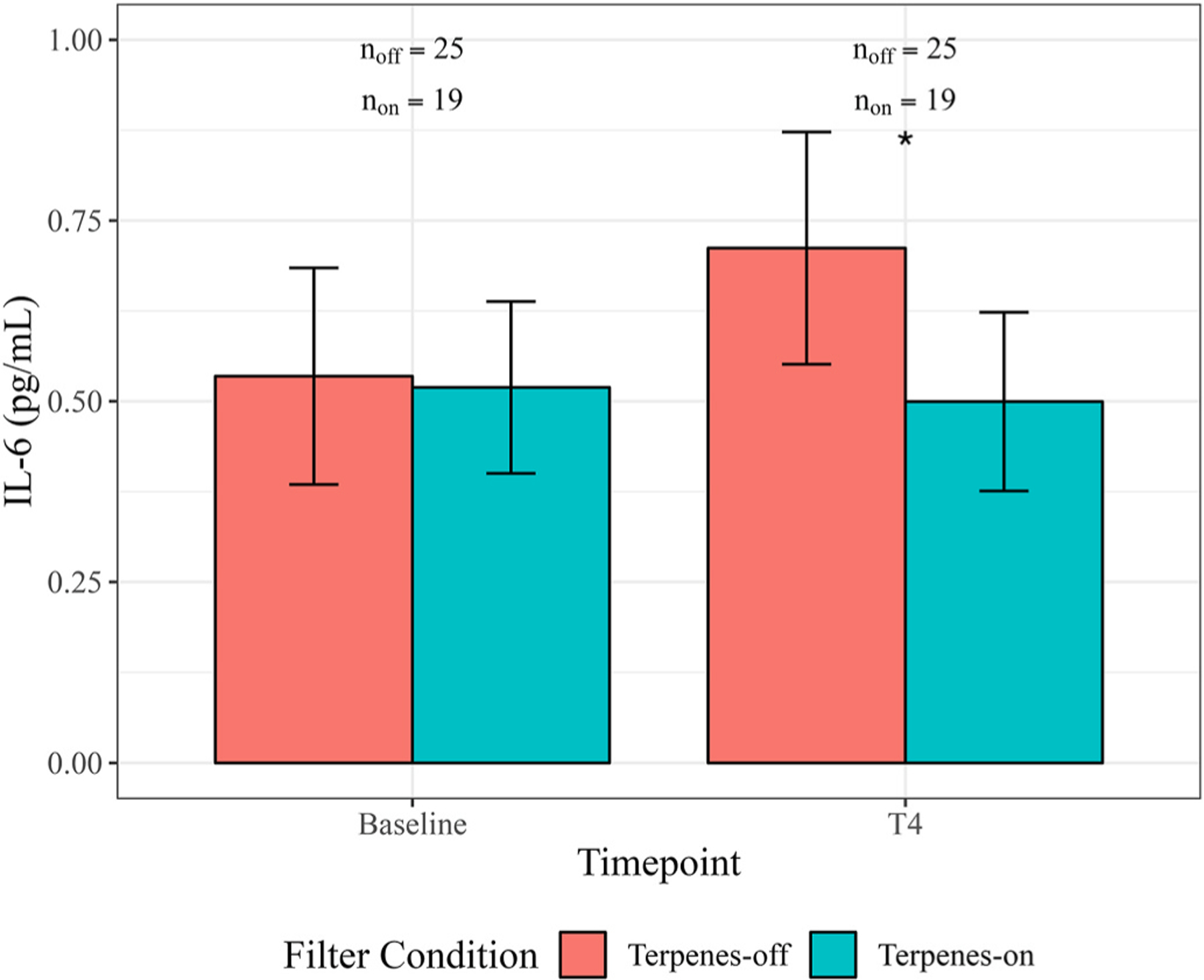
Serum IL-6 concentrations (pg/mL) at baseline and T4, for terpenes-on vs. terpenes-off conditions. Error bars represent the standard error of the mean for each filter condition group; a star represents significance at the α = 0.05 level. Sample size represents the number of IL-6 observations at each time point (baseline and T4) for each condition (terpenes-on and terpenes-off).

In the primary mixed regression analysis, we observed a statistically significant association of the terpenes-on vs. terpenes-off filter with levels of detected IL-6 in serum (see Fig. 3). T4 IL-6 concentrations were 0.19 pg/mL (95 % CI: −0.35, −0.03, *p* = 0.046) lower in the terpenes-on filter condition compared to the terpenes-off filter condition. Association estimates were similar in the sensitivity analyses using $\frac{limit\;of\;detection\;\left(LOD\right)}{\surd 2}$ or zero to impute missing values (see Supplementary Materials). The significant association of the terpenes-on vs. terpenes-off filter with IL-6 expression may have been driven by an increase in IL-6 levels in the terpenes-off condition rather than a decrease in the terpenes-on condition. However, this does not necessarily mean that the terpenes-on filter condition was not associated with anti-inflammatory activity, as it is possible that all conditions experienced some moderate stress from the blood draw or other study conditions, and the terpenes-off condition could thereby have resulted in an increase in IL-6 expression, whereas the terpenes-on condition did not. Future research should investigate this “buffering” possibility.

We observed a marginally significant association of the terpenes-on vs. terpenes-off filter with T2 levels of SCL of −0.84 μS (95 % CI: −1.73, 0.06; *p* = 0.076). Mean temperature and relative humidity at the forest site were added as additional covariates in the mixed regression model as an exploratory analysis. *P*-values changed slightly with these results (i.e., SCL *p* = 0.068, IL-6 *p* = 0.057, all other *p*-values remained insignificant; see Supplementary Materials for full results).

Additionally, we conducted a sensitivity analysis with the subset of outcome data from participants who successfully completed both sessions and for whom we had complete baseline and T2/T4 assessments. These results support our main findings, with statistically significant associations for IL-6, as well as results that are significant for SCL and TNF-α (see Table 3). T4 IL-6 concentrations were 0.18 pg/mL (95 % CI: −0.35, −0.02, *p* = 0.037) lower in the terpenes-on condition compared to the terpenes-off condition; T2 SCL values were 0.96 μS (95 % CI: −1.87, −0.05; *p* = 0.047) lower in the terpenes-on condition compared to the terpenes-off condition; and T4 TNF-α concentrations were −1.37 pg/mL (95 % CI: −2.63, −0.01, *p* = 0.045) lower in the terpenes-on condition compared to the terpenes-off condition.

### 3.2. Association of terpenes-on vs. terpenes-off filter with the pattern of outcome across entire duration of session

**Fig. 4.**
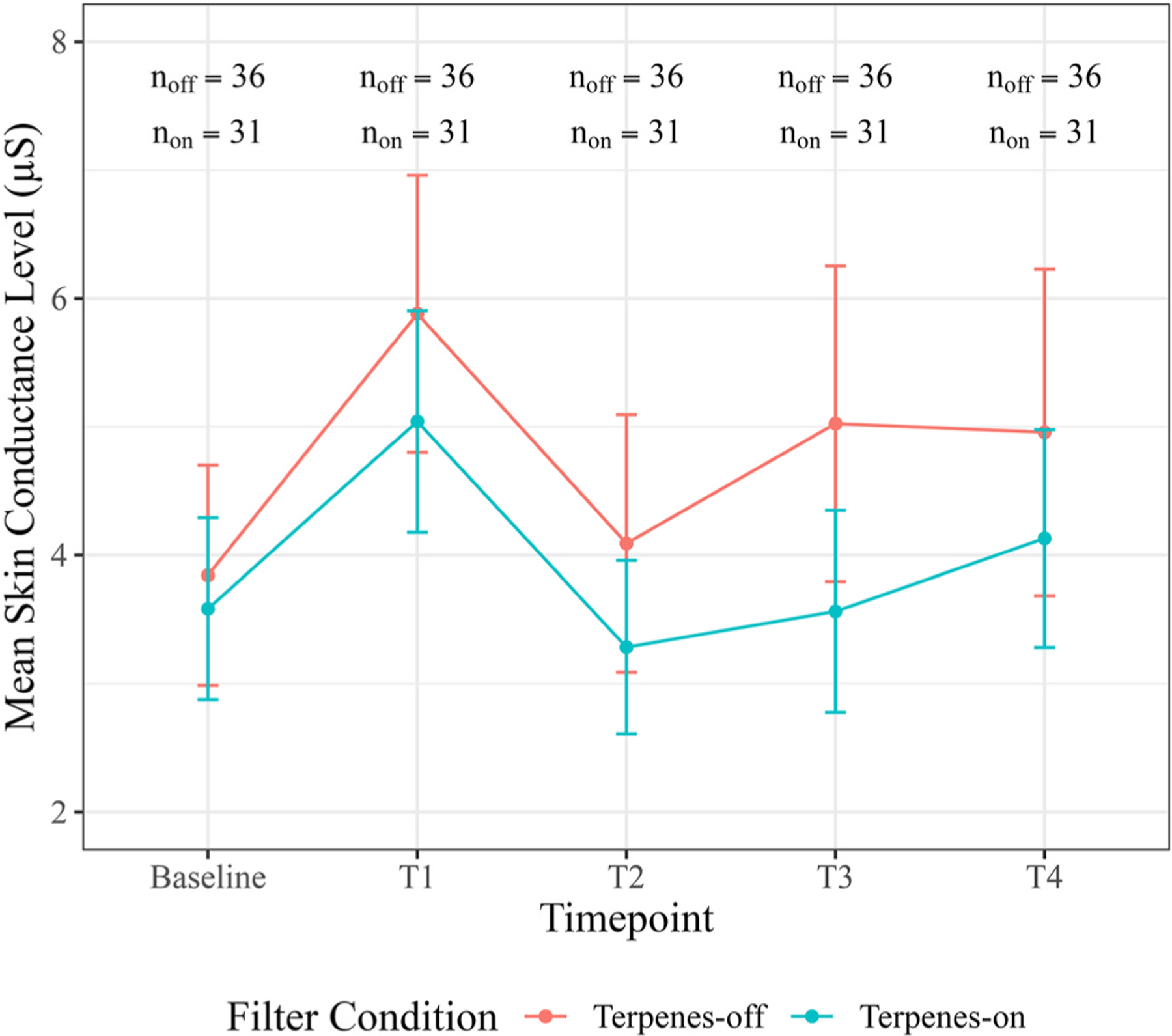
Mean skin conductance level (μS) at baseline, T1 (0–5 min of forest contact), T2 (17.5–22.5 min), T3 (37.5–42.5 min), and T4 (55–60 min), for terpenes-on vs. terpenes-off conditions. Error bars represent the standard error of the mean for each condition. Sample size represents the total number of SCL observations across all sessions at each of the time points and across both conditions (terpenes-on and terpenes-off).

In support of our hypothesis, we observed a statistically significant association of the terpenes-on vs. terpenes-off filter with the pattern of SCL taken as a whole (i.e., when assessed over the entire duration of the session) (*p* = 0.021). The terpenes-on group had 0.9 μS (95 % CI: −2.15, 0.35) lower SCL at T2, 1.56 μS (95 % CI: −2.81, −0.31) lower SCL at T3, and 0.92 μS (95 % CI: −2.17, 0.33) lower SCL at T4 compared to the terpenes-off group (see Fig. 4). All other ANOVA tests for ln-HF HRV, positive affect, negative affect, and stress were not statistically significant at the α = 0.05 level (see Table 4). Sensitivity analyses were performed by comparing model results for ln-HF HRV to model results from data with no baseline imputation, and model results from positive and negative affect outcomes to an imputed data set. Results were unchanged (see Supplementary Materials).

### 3.3. Serum terpene concentrations and absorbed dose estimates

Approximately 20 % of whole blood samples were missing (i.e., the phlebotomist/nurse was unable to draw blood samples), including nearly 50 % of T4 samples from the terpenes-on filter condition, reflecting the challenge of venipuncture sampling in a forest environment.

The differences in absorbed dose between conditions were small (see Table 5). Within these small differences, we observed negative absorbed doses in the terpenes-on sessions. In the terpenes-off sessions, we also observed negative absorbed doses of Δ-3-carene, but positive absorbed doses of α-pinene, β-myrcene, and limonene. These results may indicate confounding participant exposures to terpenes prior to our study sessions, despite our attempts to avoid this through instructions and requests for dietary and other exposures prior to sessions (see Supplemental Materials). β-pinene and β-caryophyllene absorbed doses were not included in analyses due to low detection frequencies. Estimated differences in study outcomes at T2 and T4 in association with absorbed dose are presented in Supplementary Materials. We did not observe consistent associations between absorbed dose and psychophysiological and immunological outcomes.

## 4. Discussion

To our knowledge, this investigation is the first randomized crossover trial that includes terpene filtration from ambient air, serum collection, self-reported affect, and continuous physiology assessments to examine possible associations between filtered vs. non-filtered terpenes from ambient air and correlates of stress in a forest setting. The primary aim of the study was to examine whether terpene inhalation played a role in stress reduction following exposure to ambient forest air. Additionally, we assessed whether there was an association between absorbed dose of terpenes in serum and stress outcomes.

We found no significant associations of the terpenes-on vs. terpenes-off filter with levels of ln-HF HRV, self-reported stress and affect, systolic blood pressure, diastolic blood pressure, heart rate, cortisol, TNF-α, and CRP, though we did for IL-6. The association of the terpenes-on vs. terpenes-off filter with positive affect was positive (i.e., in the hypothesized direction), but the difference between conditions was small, and confidence intervals included the null. The associations of the terpenes-on vs. terpenes-off filter with negative affect, systolic blood pressure, cortisol, TNF-α, and CRP were negative (i.e., in the hypothesized direction), but differences between conditions were small, with confidence intervals that included the null. Interestingly, the association of the terpenes-on vs. terpenes-off filter with self-reported stress, diastolic blood pressure, and heart rate were positive (i.e., against the hypothesized direction); however, the differences between conditions were also small and not statistically significant.

We found a significant association of the terpenes-on vs. terpenes-off filter with IL-6 serum levels from baseline vs. 60 min of forest contact and a significant association between the terpenes-on vs. terpenes-off filter and SCL levels taken as a whole (i.e., over the entire duration of the forest contact). These results were supported by our sensitivity analyses with the subset of data from participants who completed both conditions, which included significant associations of the terpenes-on vs. terpenes-off filter with lower levels of IL-6 at T4, and lower levels of SCL at T2 and TNF-α at T4 as well. The significant association between the terpenes-on vs. terpenes-off filter and IL-6 levels is supported by previous work, although that research did not take place in a forest setting with human participants. For example, following ischemic stroke induction in male Wistar rats, α-pinene administration significantly reduced IL-6 concentration in the hippocampus, cortex, and striatum (Khoshnazar et al., 2019). Additionally, α-pinene has been associated with a significant reduction in IL-6 production in mouse peritoneal macrophages following lipopolysaccharide-induced inflammation, and with decreased IL-6 expression in male Wistar rats following isoproterenol (ISO)-induced inflammation (Kim et al., 2015; Zhang et al., 2020).

In this case, our results indicate that the significant association may have been driven more by an increase of IL-6 expression in the terpenes-off condition than a decrease in the terpenes-on condition. However, this does not necessarily indicate a lack of an association of the terpenes-on condition with anti-inflammatory activity, as moderate stress associated with some aspects of the study protocol (e.g., blood draws) may have resulted in elevated inflammatory and physiological markers in the terpenes-off condition, while the terpenes-on condition may have helped to buffer against this increase – thereby remaining relatively stable in terms of total values from pre-to post-session. We do not know if this is the case, however, and future research should investigate this possibility.

The association of the terpenes-on vs. terpenes-off filter with skin conductance levels at T2 was negative and in the hypothesized direction, but only marginally significant (though significant in the subset of data used for sensitivity analyses). The association with the pattern of skin conductance level response over the course of the entire forest contact sessions was statistically significant, and the effect estimate at each time point was negative and in the hypothesized direction (though confidence intervals crossed zero at the individual timepoints of T2 and T4).

Though they should be considered with caution and substantial limits to interpretation, our trend-level findings in the hypothesized directions for positive affect, negative affect, systolic blood pressure, cortisol, TNF-α, and CRP are generally in line with those posited from the Shinrin-yoku literature (Antonelli et al., 2020; Park et al., 2009). Our trend-level results for self-reported stress, diastolic blood pressure, and heart rate that were against our hypothesized directions are not representative of typical findings in the literature. For example, though it employed a different design and did not take place in a forest setting, a study that includes humans found significant decreases in heart rate and diastolic blood pressure following fir essential oil exposure and significant increases in HRV following aromatherapy exposure (e.g., Chang and Shen, 2011). However, other studies of essential oil or terpene exposure in a controlled setting have similar results to ours insofar as they lack significant findings, including, for example, no significant differences in HR and CRP levels when comparing groups of people exposed to essential oils versus controls (Jun et al., 2013). These results may also reflect the complexity and inconsistency with which physiological indicators sometimes track with subjective experiences of stress.

Nature contact represents an interaction between human and environmental systems, and a contextual and subjective encounter for an individual that includes a combination of diverse sensory, psychological, and physiological experiences. Individuals may process sensory stimuli differently and vary in their subjective experiences and tendencies to attend to different aspects or elements of their environments (Bratman et al., 2021; Kitayama et al., 2003; Partos et al., 2016; Simmons and Estes, 2008; White and Burton, 2022). This experiment was designed to focus on one feature of an experience with nature (i.e., terpene inhalation), and did not consider the diverse array of other nature interactions that can be experienced by an individual.

### 4.1. Limitations and future directions

Our study had several limitations. First, our target sample size was 40 participants, and while we enrolled 43 people, only 31 participants completed both sessions, preventing us from being fully powered for our within-subjects analytic design. Additionally, missing blood samples and small amounts of missing physiology data introduced a potential source of sampling bias by further reducing the sample size and potentially limiting the generalizability of our findings. With N = 40, we were powered to detect an expected mean 5 % reduction in heart rate (Hedges’ g = 0.4), 2 % reduction in blood pressure (Hedges’ g = 0.17), 35 % increase in high frequency heart rate variability (Hedges’ g = 0.12), and 32 % decrease in self-reported tension and anxiety. It should be noted that our target sample size was based on studies that observed differences in stress-related outcomes when comparing different environments (e.g., forest vs. urban contact conditions), and not studies that compared terpene inhalation within the same natural environment (e.g., terpenes-on vs. terpenes-off conditions), as these were not available. Therefore, we may have been underpowered compared to the effect sizes reported in previous studies under different conditions.

Several factors impacted participant attrition, including lack of available study session days due to wildfire smoke, heat conditions, and the limitation of sampling windows to June through September due to precipitation and temperature conditions in the U.S. Pacific Northwest area during the rest of the year. Additionally, the randomly generated participant arm assignment led to a greater number of BA participants being enrolled during the first summer, when wildfire smoke and high heat events had a greater impact on the need for session rescheduling and cancellation and subsequent participant attrition. This imbalance in treatment group completion may reduce the generalizability of our findings, along with sample characteristics, such as a relatively high proportion of participants without current employment or who preferred not to answer this question. Attrition issues were addressed following the first summer of sampling by installing air conditioning in study rooms to increase available sampling days and by a small increase in participant compensation.

Second, this study differs from other forest bathing studies as we intentionally did not include a guided element that instructed participants to pay attention to sensory experiences, including odors, due to our intention to examine ecologically valid conditions in which forest contact occurs without specific instructions (Clarke et al., 2021; Li, 2022; Satyawan et al., 2022). As a result of this lack of instruction, participants may not have attended to forest smells, potentially moderating psychological responses and odor perception (Oleszkiewicz et al., 2021). Preliminary smell perception and experience results suggest that filter conditions were quite similar with respect to subjective experience of forest smells (see Supplemental Material). This indicates that participants may not have been able to explicitly perceive terpenes when they were exposed to them, potentially preventing some psychological responses and conscious appraisals of odors. Because participants were blinded to condition and consistently breathing some form of filtered air via PAPR masks during each session, our goal was to avoid priming from expectations regarding the effects of odors.

Third, we did not observe consistent differences in absorbed dose between filter conditions, in contrast to a previous study that found serum α-pinene increases following 1 h of forest walking (n = 4) (Sumitomo et al., 2015). However, this conclusion should be considered within the context of the fact that our sample size was limited with respect to serum data. Additionally, in line with our findings, Bach et al. (2021) observed no significant difference in monoterpene concentrations in blood between groups after 2-h exposures to a forest vs. urban environment.

In the current study, there was no clear pattern in serum concentrations that indicated changes in absorbed dose of terpenes, and some participants may have experienced a decay of terpene serum concentrations, potentially due to metabolism of terpenes that were present in their circulation prior to the forest bathing session (Falk et al., 1990). Although we asked participants to avoid certain foods, beverages, and products in the 24 h before each session, this relied on participant compliance. Future studies may try to control diet and product exposure for a longer period by providing meals and lodging before each experience, in order to keep baseline serum terpene concentrations consistent. As with our results resulting from serum analyses, these findings may also be due to the limitations in our sample size that came from challenges of collecting serum in the forest setting. Alternatively, it may indicate that the associations and trends we observed in psychophysiological and immunological outcomes were more directly relevant to olfactory receptor and subsequent neural activity vs. absorption in the blood following inhalation. We note that the sample size was larger for other outcomes, such as skin conductance, and we did not have a complete set of absorbed dose data with which to calculate associations with physiological outcomes, so further research is needed on these questions.

Fourth, though included intentionally and as a strength of our experimental design with respect to controlling potentially confounding factors, participants had limited interaction with the forest beyond visual and olfactory exposures. Participants were seated for 1 h while wearing the PAPR helmet, which limited other sensory interactions (e.g., tactile or auditory interaction) that are explored elsewhere in the nature and health literature (Bratman et al., 2019).

Finally, for the statistically significant associations that we did observe for IL-6 and skin conductance, our study design did not allow for a differentiation between potential causal effects mediated by terpene binding with olfactory receptor neurons vs. effects mediated by terpene influence on biochemical pathways following lung, circulatory (e.g., passing the blood brain barrier), skin, or gastrointestinal uptake and absorption. Future research should further investigate the relevance of these pathways in human health.

## 5. Conclusion

This study examined the association of inhaling ambient forest air that was filtered vs. non-filtered from terpenes with correlates of stress in adults, using a within-subjects randomized-crossover design. We found no significant associations of the terpenes-on vs. terpenes-off filter with high frequency heart rate variability, positive affect, negative affect, self-reported stress, blood pressure, heart rate, TNF-α, CRP, and cortisol at specific time points. However, we did observe a significant association of the terpenes-on vs. terpenes-off filter with levels of IL-6 in serum before vs. after the session, and with skin conductance as a whole (i.e., over the entire duration of the session). Additionally, the trend-level associations between the terpenes-on vs. terpenes-off filter and positive affect, negative affect, systolic blood pressure, cortisol, TNF-α, and CRP were all in the hypothesized direction. Sensitivity analyses on the subset of observations from participants who completed sessions in both conditions support these trends as well, including significant associations of the terpenes-on vs. terpenes off filter with levels of IL-6, SCL, and TNF-α.

The pattern of findings of associations between the terpenes-on vs. terpenes-off filter and study outcomes may indicate that our pilot study was underpowered to adequately assess statistical significance with a sample size of 40. Future research should further explore these results with larger sample sizes. It should also be noted that we had high levels of missingness and non-detect observations for serum terpene concentrations that prevented us from being fully powered to examine associations between terpene absorbed dose and the target psychophysiological outcomes or the association between the terpenes-on vs. terpenes-off filter and levels of inflammatory biomarkers and cortisol.

This pilot study was designed to focus on a particular aspect of nature contact – terpene inhalation in a real-world setting – and aimed to control for confounding variables via a rigorous experimental design. The work is a starting point for future exploration into the multi-sensory aspects of nature experience and one of the many potential pathways and mechanisms that could explain the positive health benefits of nature contact. This research should be contextualized in the broader nature and health literature as a step forward in examining and isolating a potential pathway, but not an attempt to explain all well-being changes following different types of nature contact. Future research should continue to investigate the relationship between terpene exposure – particularly at naturally occurring ambient levels – and human health.

## Supplementary Material

MMC1

## Figures and Tables

**Table 1:** Baseline characteristics and demographic information of study participants by sequence.

	Sequence AB (N = 21)	Sequence BA (N = 22)	Overall (N = 43)
**Age (years)**			
Mean (SD)	33.0 (14.2)	37.1 (14.5)	35.1 (14.3)
Median [Min, Max]	28.0 [20.0, 65.0]	35.5 [19.0, 68.0]	29.0 [19.0, 68.0]
**Gender**			
Male	7 (33.3 %)	10 (45.5 %)	17 (39.5 %)
Female	12 (57.1 %)	10 (45.5 %)	22 (51.2 %)
Prefer to self-describe	1 (4.8 %)	1 (4.5 %)	2 (4.7 %)
Non-binary	1 (4.8 %)	1 (4.5 %)	2 (4.7 %)
**Race**			
American Indian or Alaska Native	0 (0 %)	1 (4.5 %)	1 (2.3 %)
Asian	2 (9.5 %)	3 (13.6 %)	5 (11.6 %)
Black or African American	1 (4.8 %)	2 (9.1 %)	3 (7.0 %)
Native Hawaiian or Other Pacific Islander	0 (0 %)	0 (0 %)	0 (0 %)
White	16 (76.2 %)	15 (68.2 %)	31 (72.1 %)
Other	2 (9.5 %)	1 (4.5 %)	3 (7.0 %)
**Ethnicity**			
Hispanic, Latino or Spanish	2 (9.5 %)	3 (13.6 %)	5 (11.6 %)
Non-Hispanic, Latino or Spanish	19 (90.5 %)	19 (86.4 %)	38 (88.4 %)
**Education**			
High school graduate (high school diploma or equivalent including GED)	1 (4.8 %)	3 (13.6 %)	4 (9.3 %)
Some college but no degree	5 (23.8 %)	1 (4.5 %)	6 (14.0 %)
Associate degree in college (2-year)	1 (4.8 %)	1 (4.5 %)	2 (4.7 %)
Bachelor’s degree in college (4-year)	11 (52.4 %)	11 (50.0 %)	22 (51.2 %)
Graduate degree or higher	3 (14.3 %)	6 (27.2 %)	9 (20.9 %)
**Income**			
Less than $19,999	2 (9.6 %)	1 (4.5 %)	3 (7.0 %)
$20,000 to $39,999	9 (42.8 %)	4 (18.2 %)	13 (30.3 %)
$40,000 to $59,999	3 (14.3 %)	3 (13.6 %)	6 (14.0 %)
$60,000 to $79,999	0 (0 %)	3 (13.6 %)	3 (7.0 %)
$80,000 to $99,999	1 (4.8 %)	0 (0 %)	1 (2.3 %)
$100,000 to $149,999	1 (4.8 %)	7 (31.8 %)	8 (18.6 %)
$150,000 to $299,999	2 (9.5 %)	2 (9.0 %)	4 (9.3 %)
$300,000 or more	2 (9.5 %)	0 (0 %)	2 (4.7 %)
Prefer not to answer	1 (4.8 %)	2 (9.1 %)	3 (7.0 %)
**UPSIT Score**			
Mean (SD)	33.6 (2.56)	32.1 (3.19)	32.8 (2.96)
Median [Min, Max]	35.0 [28.0, 37.0]	33.0 [25.0, 37.0]	33.0 [25.0, 37.0]
**Baseline Perceived Stress Score (PSS)**			
Mean (SD)	7.76 (1.51)	8.09 (1.15)	7.93 (1.33)
Median [Min, Max]	8.00 [4.00, 11.0]	8.00 [6.00, 10.0]	8.00 [4.00, 11.0]
**Nature Relatedness Scale**			
Mean (SD)	4.17 (0.593)	4.30 (0.617)	4.24 (0.601)
Median [Min, Max]	4.33 [2.50, 5.00]	4.50 [2.83, 5.00]	4.50 [2.50, 5.00]
**Nature Frequency**			
Once a month	1 (4.8 %)	1 (4.5 %)	2 (4.7 %)
2–3 times a month	1 (4.8 %)	1 (4.5 %)	2 (4.7 %)
Once a week	3 (14.3 %)	1 (4.5 %)	4 (9.3 %)
2–3 days a week	8 (38.1 %)	7 (31.8 %)	15 (34.9 %)
4–5 days a week	3 (14.3 %)	8 (36.4 %)	11 (25.6 %)
6–7 days a week	5 (23.8 %)	4 (18.2 %)	9 (20.9 %)
**Nature Duration (hours/week)**			
Mean (SD)	13.4 (13.6)	16.4 (19.9)	14.9 (16.9)
Median [Min, Max]	10.0 [3.00, 50.0]	12.0 [5.00, 100]	10.0 [3.00, 100]
Missing	0 (0 %)	1 (4.5 %)	1 (2.3 %)

**Table 2:** Association of the terpenes-on vs. terpenes-off filter with study outcomes at T2 (20 min) or T4 (60 min).

Outcome	Observed Time Point	Terpenes-on Parameter Estimate	95 % CI	Sample Size (participant sessions)	*p*
ln-HF HRV	T2	0.01	(−0.18, 0.21)	59	0.914
SCL	T2	−0.84	(−1.73, 0.06)	67	0.076
PA	T2	0.70	(−0.37, 1.77)	66	0.210
NA	T2	−0.16	(−0.43, 0.12)	66	0.265
Stress	T2	0.01	(−0.19, 0.21)	56	0.921
DBP	T4	1.13	(−2.58, 4.85)	67	0.554
SBP	T4	−0.53	(−6.06, 5.00)	67	0.852
HR	T4	0.13	(−1.93, 2.18)	67	0.905
Cortisol	T4	−2.85	(−17.95, 12.24)	44	0.716
IL-6	T4	−0.19	(−0.35, −0.03)	44	**0.046** *
TNF-α	T4	−0.79	(−1.88, 0.31)	44	0.166
CRP	T4	−0.34	(−2.2, 1.52)	44	0.724

* *p* < 0.05.

Note: ln-HF HRV = ln high frequency heart rate variability; SCL = skin conductance level; PA = positive affect; NA = negative affect; DBP = diastolic blood pressure; SBP = systolic blood pressure; HR = heart rate. Associations of the terpenes-on vs. terpenes-off filter were assessed using ln HF HRV, SCL, PA, NA, and stress at time point 2 (i.e., T2) and DBP, SBP, HR, cortisol, IL-6, TNF-α, and CRP at time point 4 (i.e., T4).

**Table 3:** Association of the terpenes-on vs. terpenes-off filter with study outcomes at T2 (20 min) or T4 (60 min) for participants with complete session 1 and session 2 baseline and observed timepoint outcome.

Outcome	Observed Time Point	Terpenes-on Parameter Estimate	95 % CI	Sample Size (participant sessions)	*p*
ln HF-HRV	T2	0.02	(−0.18, 0.23)	50	0.815
SCL	T2	−0.96	(−1.87, −0.05)	60	**0.047** *
PA	T2	0.58	(−0.54, 1.70)	60	0.318
NA	T2	−0.18	(−0.47, 0.12)	60	0.242
Stress	T2	−0.04	(−0.27, 0.18)	46	0.710
DBP	T4	1.79	(−2.16, 5.73)	58	0.379
SBP	T4	0.47	(−5.61, 6.55)	58	0.880
HR	T4	0.88	(−1.20, 2.96)	58	0.413
Cortisol	T4	−3.80	(−20.66, 13.06)	26	0.669
IL-6	T4	−0.18	(−0.35, −0.02)	26	**0.037** *
TNF-α	T4	−1.37	(−2.63, −0.10)	26	**0.045** *
CRP	T4	−0.56	(−1.97, 0.84)	26	0.448

* *p* < 0.05.

Note: ln-HF HRV = ln high frequency heart rate variability; SCL = skin conductance level; PA = positive affect; NA = negative affect; DBP = diastolic blood pressure; SBP = systolic blood pressure; HR = heart rate. Associations of the terpenes-on vs. terpenes-off filter were assessed using ln HF HRV, SCL, PA, NA, and stress at time point 2 (i.e., T2) and DBP, SBP, HR, cortisol, IL-6, TNF-α, and CRP at time point 4 (i.e., T4).

**Table 4:** ANOVA results comparing full and reduced models to test the association of the terpenes-on vs. terpenes-off filter with patterns of outcome over the session as a whole.

Model	AIC	BIC	Log Likelihood	Deviance	Chisq	df	*p*
**ln-HF HRV**							
Reduced	296.26	320.42	−141.13	282.26			
Full	300.11	338.07	−139.06	278.11	4.15	4	0.386
**SCL**							
Reduced	1366.73	1391.86	−676.36	1352.73			
Full	1363.20	1402.70	−670.60	1341.20	11.53	4	**0.021**
**Positive Affect**							
Reduced	1280.38	1305.49	−633.19	1266.38			
Full	1285.45	1324.91	−631.73	1263.45	2.93	4	0.569
**Negative Affect**							
Reduced	575.95	601.06	−280.97	561.95			
Full	581.35	620.81	−279.68	559.35	2.59	4	0.628
**Stress**							
Reduced	301.63	325.60	−143.81	287.63			
Full	308.11	345.78	−143.05	286.11	1.52	4	0.823

Note: ln-HF HRV = ln high frequency heart rate variability; SCL = skin conductance level; DBP = diastolic blood pressure; SBP = systolic blood pressure; HR = heart rate; AIC = Akaike information criterion; BIC = Bayesian information criterion.

**Table 5:** Reporting limits, detection frequencies, and concentrations of 6 terpenes in serum and air samples from forest sitting sessions.

Terpene		Forest Air (n = 31 sampling days)			Baseline Serum (n = 41 sessions)^a^			Follow-up Serum, Terpenes-on (n = 17 sessions)				Follow-up Serum, Terpenes-off (n = 24 sessions)			
	LOD (μg/L)	%≥ MRL	MRL (ng)	50th perc. (ng/m3)	% ≥ LOD	50th perc. (μg/L)	95th perc. (μg/L)	% ≥ LOD	50th perc. (μg/L)	95th perc. (μg/L)	Abs. Dose Mean (SD) (μg/L)	% ≥ LOD	50th perc. (μg/L)	95th perc. (μg/L)	Abs. Dose Mean (SD) (μg/L)
α-pinene	0.050	71	0.25–13	149	56	0.05	0.09	53	0.05	0.07	−0.003 (0.02)	71	0.07	0.11	0.006 (0.03)
β-pinene	0.056	71	0.25–2.7	84	44	–	0.15	35	–	0.12	–	63	0.07	0.15	–
β-myrcene	0.085	48	0.25–1.0	–	90	0.16	0.25	100	0.17	0.23	−0.003 (0.06)	79	0.16	0.25	0.001 (0.07)
Δ-3-carene	0.032	42	0.25–2.0	–	93	0.05	0.08	88	0.05	0.07	−0.001 (0.01)	88	0.04	0.12	−0.005 (0.03)
limonene	0.162	39	0.25–3.4	–	98	0.50	1.9	100	0.34	0.89	−0.032 (0.09)	100	0.63	2.8	0.007 (0.13)
β-caryophyllene & α-cedrene (air)/β-caryophyllene (serum)^b^	0.600	29	0.25–4.0	–	17	–	–	6	–	–	–	17	–	–	–

Note: LOD = limit of detection. Perc. = percentile; lower percentiles not reported for analytes detected in fewer than 50 % of samples. Abs. Dose = absorbed dose; MRL = method reporting limit; MRLs set at highest mass detected in any blank during that period; maximum likelihood estimation (MLE) was used to impute non-detect observations for α-pinene, β-myrcene, and Δ-3-carene to calculate absorbed dose. Due to a low proportion of non-detect observations, $\frac{\text{LOD}}{\sqrt{2}}$ was used to impute non-detect observations for limonene absorbed dose. Due to high proportions of non-detects, no imputation was performed on β-pinene and β-caryophyllene data.

a 19 participants contributed one sample each while 11 contributed two samples each.

b β-caryophyllene & α-cedrene could not be resolved in the air analyses. Number of sessions refers to the number of individual terpenes-on or terpenes-off filter condition sessions.

## Data Availability

Data will be made available on request.
