# Supplementary material for: Forest terpenes and stress: Examining the associations of filtered vs. non-filtered air in a real-life natural environment
Source: Environ Res. Author manuscript; Available in PMC 2025 Jul 1. (PMC12103269; doi:10.1016/j.envres.2025.121482)
Supplement: MMC1 [file NIHMS2071347-supplement-MMC1.pdf]

Supplementary Materials

1. Products to avoid 24 hours before forest-sitting session

- Consumer products
  - Scented body lotions, shampoos, conditioners
  - Essential oil treatments of any kind, for example, oils or sprays
  - Air fresheners or diffusers
  - Anything with basil, black pepper, cardamom, cedar, citrus, eucalyptus, juniper, lavender, pine, rosemary, thyme, cloves, mint, or sage scent
  - Products containing tea tree oil
  - Natural insect repellants
  - Vicks® VapoRub™ or similar over-the-counter drugs, rubs, or inhalers
- Cleaning products
  - Any scented cleaning product, except for vinegar, water, or household bleach
  - Anything with basil, black pepper, cardamom, cedar, citrus, eucalyptus, juniper, lavender, pine, rosemary, thyme, cloves, mint, or sage scent
  - Floor wax
  - Turpentine
- Marijuana and e-cigarettes
- Non-steroidal anti-inflammatory drugs (NSAIDs): any non-steroidal anti-inflammatory drugs, including aspirin, ibuprofen (like Motrin® or Advil®), and naproxen sodium (like Aleve®)
- Food and Drink

- Orange, grapefruit, and any other citrus-based juices (including a squeeze of lemon or lime)
- Beer, gin, wine, and other alcoholic drinks (including mixers like tonic water, and lemon or lime twists)
- Beverages flavored with cardamom, juniper, lavender, pine, rosemary, thyme, cloves, mint, or sage
- Citrus fruits, recipes, or processed foods heavy on the following herbs (dried or fresh): black pepper, basil, caraway, cardamom, coriander, fennel, juniper berry, rosemary, sage, thyme, lavender, cloves; chewing gum or herbal candy or cough drops.

2. Power Calculation

The estimated numbers needed to detect minimum differences in HF-HRV in the contrasting conditions for the same participant were determined based on the minimums in the ranges of Hedges' g values from prior studies and standard population-level intra-individual differences.

3. Average Nature Contact Duration, Frequency, and Relatedness

Participants' usual nature contact was assessed using measures from Bratman et al. (2024) and Bratman et al. (2021), based on operationalizations from other nature exposure papers (Shanahan et al., 2016; M. P. White et al., 2019). Average nature contact frequency was assessed by presenting participants with the following question: "About how often do you usually visit or pass through outdoor natural areas for any reason? This includes, for example, walking, biking, or recreating outside in local, regional, or national parks, at the beach, beside or within lakes, creeks, or the ocean, gardening or tending to plants, camping, fishing, reading or walking outside next to trees, engaging in yard work with natural elements, etc…" Participants selected an

answer from following options: “Never”, “Once a year”, “Once every three months”, “Once a month”, “2-3 times a month”, “Once a week”, “2-3 days a week”, “4-5 days a week”, or “6-7 days a week” (Bratman et al., 2024). Average nature contact duration was measured by presenting participants with the following question: “Over the last month, approximately how many HOURS PER WEEK do you consider yourself to have interacted with nature? This includes, for example, walking, biking, or recreating outside in local, regional, or national parks, at the beach, beside or within lakes, creeks, or the ocean, gardening or tending to plants, camping, fishing, reading or walking outside next to trees, engaging in yard work with natural elements, etc...” (Bratman et al., 2021). Participants entered their response in a provided text box.

Nature relatedness was measured during baseline measurements using the short-form nature relatedness scale (NR-6; Nisbet & Zelenski, 2013). The NR-6 consists of 6 items designed to capture how people view their relationship with nature (e.g., “My relationship to nature is an important part of who I am”). Each item is rated on a 5-point scale, ranging from 1 (“Disagree strongly”) to 5 (“Agree strongly”) with a total score calculated by averaging all six items. Cronbach’s α was used to measure how closely correlated related scale items were as a group. In the current study, NR-6 showed good reliability (Cronbach’s α = .76).

General levels of affect (PANAS-20) reported here:

	Terpenes-on	Terpenes-off	Overall
**Positive Affect (PANAS-20)**			
Mean (SD)	31.9 (6.02)	35.2 (6.52)	33.6 (6.43)
Median [Min, Max]	34.0 [20.0, 40.0]	37.0 [21.0, 45.0]	35.0 [20.0, 45.0]
**Negative Affect (PANAS-20)**			
Mean (SD)	19.5 (6.40)	16.4 (5.21)	17.9 (5.96)
Median [Min, Max]	17.0 [11.0, 30.0]	16.0 [10.0, 26.0]	17.0 [10.0, 30.0]

4. Baseline HRV Imputation Approach

Additional 5 min periods of ln-HF HRV were calculated using continuous data from before treatment exposure, including 10-5 min before B1 (R1), 5-0 minutes before B1 (R2), 0-5 min into the venipuncture procedures (V1), 5-10 min into the venipuncture procedure (V2), 0-5 min into the drive up to the forest site (D1), and 5-10 min into the drive up to the forest site (D2). Correlations between B1 ln-HF HRV and each additional time point before exposure were assessed using a complete case data set. Time points with the highest correlations (R1, R2, V1, D1) were included in single linear regression models to predict B1 values using a complete case data set. One participant did not have any additional time points available, so we used a regression model with the B1 measurement from the other session as a predictor (s2_b1_hrv ~ s1_b1_hrv). Each single linear regression model (b1_hrv ~ r1_hrv, b1_hrv ~ r2_hrv, b1_hrv ~ v1_hrv, b1_hrv ~ d1_hrv, s2_b1_hrv ~ s1_b1_hrv) was cross-validated using a complete-case dataset to assess R^2 values (0.92, 0.95, 0.98, 0.85, and 0.83, respectively). Models were preferentially ranked based on R^2 values and predicted values for B1 were imputed depending on R1, R2, V1, D1, and S1B1 (session 1 baseline 1) timepoint availability. Of the missing baseline observations, there is one person for whom we used V1 to predict B1, three people for whom we used D1 to predict B1, and one person for whom we used S1B1 to predict S2B1.

5. Statistical Models to Evaluate the Effect of Forest Terpenes Filter on the Pattern of Outcomes Across Entire Duration of Session

Null model:

$$Y_{its} = \beta_0 + \beta_1 baseline_{is} + \beta_2 T2_{is} + \beta_3 T3_{is} + \beta_4 T4_{is} + \alpha_i + \varepsilon_{its} \qquad (2)$$

where Y_{its} is the observed mean outcome for participant *i*, at time point *t* (T1, T2, T3, or T4), and session *s* (session 1 or session 2), β_0 is the value of Y_{its} at T1 and adjusted for baseline, β_1 is the estimated difference in Y_{its} at T1, T2, T3, or T4 between two groups differing in baseline by 1

unit, β_2 is the difference between Y_{its} comparing T1 and T2, β_3 is the difference between Y_{its} comparing T1 and T3, β_4 is the difference between Y_{its} comparing T1 and T4, α_i is the random intercept, and ε_{its} is the observation-specific error.

Full model:

$$Y_{its} = \beta_0 + \beta_1 baseline_{is} + \beta_2 T2_{is} + \beta_3 T3_{is} + \beta_4 T4_{is} + \beta_5 filter_{is} + \beta_6 T2_{is} * filter_{is} + \beta_7 T3_{is} * filter_{is} + \beta_8 T4_{is} filter_{is} + \alpha_{is} + \varepsilon_{its} \quad (3)$$

where Y_{its} is the observed mean outcome for participant *i*, at time point *t* and session, β_0 is the mean Y_{its} at T1 and adjusted for baseline for the study population when they were assigned the "terpenes-off" filter, β_1 is the estimated difference in Y_{its} at T1, T2, T3, or T4 between two groups differing in baseline by 1 unit, β_2 is the average difference in Y_{its} comparing T1 and T2 for the study population when they were assigned the "terpenes-off" filter, adjusting for baseline, β_3 is the average difference in Y_{its} comparing T1 and T3 for the study population when they were assigned the "terpenes-off" filter, adjusting for baseline, β_4 is the average difference in Y_{its} comparing T1 and T4 for the study population when they were assigned the "terpenes-off" filter, adjusting for baseline, β_5 is the average difference between Y_{its} at T1 when the study population was assigned the "terpenes-off" filter and Y_{its} at T1 when they were assigned the "terpenes-on" filter, adjusted for baseline, β_6 is the average difference between Y_{its} at T1 and T2 when the study population was assigned the "terpenes-on" filter, adjusted for baseline, β_7 is the average difference between Y_{its} at T1 and T3 when the study population was assigned the "terpenes-on" filter, adjusted for baseline, β_8 is the average difference between Y_{its} at T1 and T4 when the study population was assigned the "terpenes-on" filter, adjusted for baseline, α_i is the random intercept for subjects to account for repeated measures within individuals, and ε_{its} is the observation-specific error.

For positive affect and negative affect, a parameter was added to each model to adjust for missing scale items (items left unanswered on the I-PANAS-SF).

6. PAPR Mask and Filter Car Filtration Results

PAPR effectiveness was evaluated under laboratory conditions by generating a controlled atmosphere of ~ 40 ppm terpinolene, pumping that atmosphere into the PAPR helmet via the PAPR blower, then measuring the terpinolene concentrations inside the PAPR helmet using a photoionization detector. In this setting the charcoal filter (Filter B) reduced terpinolene concentrations relative to the HEPA filter (Filter A) by 98±0.1% (n=4 trials).

We also evaluated PAPR effectiveness in the forest setting by sampling ambient terpene concentrations inside the PAPR helmet using the thermal desorption tubes, with either the A or B filter installed. We observed an average 68% reduction (SD=26% in α-pinene concentrations and 80% reduction (SD=24%) in β-pinene concentrations from the "terpenes-off" filter (Filter B) compared to the "terpenes-on" filter (Filter A). Variability in our measurements of filter effectiveness in the forest likely represents measurement error (i.e. variability in our experimental sampling and analysis procedures for measuring terpenes), rather than true variability in filter effectiveness, as laboratory testing after data collection indicated that the terpenes-off filters were consistently effective.

We also tested the effectiveness of the in-vehicle filtration system by measuring terpene concentrations within the vehicle while driving between Tacoma and Pack Forest, with the in-vehicle filter either on or off. Across 10 measurement events we observed an average 91% reduction in α-pinene concentrations and 90% reduction in β-pinene concentrations when the filter was on.

7. Temperature and Relative Humidity Controlled in Analyses

During seated forest sessions, the mean temperature was 19.7°C (SD = 4.19°C) and had a range from 12.4°C to 31.5°C. For relative humidity, the mean was 61.6% (SD=14.1%) and had a range from 32.0% to 94.0%.

Table 1. Effect of terpene exposure on study outcomes at T2 (20 min) or T4 (60 min), with additional covariates of mean temperature and relative humidity.

Outcome	Observed Time Point	Terpenes-on Exposure Parameter Estimate	95% CI	*p*
ln HF-HRV	T2	-0.01	(-0.2,5)	0.929
SCL	T2	-0.85	(-1.73,4.62)	0.068
PA	T2	0.72	(-0.33,3.36)	0.189
NA	T2	-0.14	(-0.42,6.96)	0.327
Stress	T2	0.01	(-0.19,1.69)	0.904
DBP	T4	1.02	(-2.74,61.78)	0.596
SBP	T4	-0.8	(-6.37,82.74)	0.778
HR	T4	0.25	(-1.72,15.47)	0.809
Cortisol	T4	-4.37	(-20.26,79.48)	0.599
IL-6	T4	-0.19	(-0.36,1.99)	0.057
TNF-α	T4	-0.75	(-1.86,10.23)	0.195
CRP	T4	0.05	(-1.75,15.41)	0.961

Imputation Results:

Table 2. Imputed affect and baseline ln-HF HRV T2 sensitivity analysis results.

Model		Estimate	95% CI	*p*
ln-HF HRV				
	No Imputation	0.00	(-0.21, 0.20)	0.967
	Imputation	0.01	(-0.18, 0.21)	0.914
Positive Affect				
	No Imputation	0.70	(-0.37, 1.77)	0.210
	Imputation	0.71	(-0.36, 1.77)	0.202
Negative Affect				
	No Imputation	-0.16	(-0.43, 0.12)	0.265
	Imputation	-0.16	(-0.42, 0.11)	0.250
IL-6				
	No Imputation	-0.19	(-0.35, -0.03)	**0.046**
	$\frac{LOD}{\sqrt{2}}$ Imputation	-0.18	(-0.34, -0.02)	0.051
	Zero Imputation	-0.19	(-0.36, -0.02)	0.050

Note: ln-HF HRV = ln high frequency heart rate variability.

Table 3. Imputed affect and baseline ln-HF HRV ANOVA comparing full and reduced models.

Model	Model Type	AIC	BIC	Log Likelihood	Deviance	Chisq	df	*p*
**ln-HF HRV**								
Imputed	Reduced	296.26	320.42	-141.13	282.26			
	Full	300.11	338.07	-139.06	278.11	4.15	4	0.386
Not Imputed	Reduced	266.07	289.60	-126.03	252.07			
	Full	272.12	309.09	-125.06	250.12	1.95	4	0.745
**Positive Affect**								
Not Imputed	Reduced	1280.38	1305.49	-633.19	1266.38			
	Full	1285.45	1324.91	-631.73	1263.45	2.93	4	0.569
Imputed	Reduced	1289.37	1314.50	-637.68	1275.37			

Model	Model Type	AIC	BIC	Log Likelihood	Deviance	Chisq	df	*p*
	Full	1294.48	1333.98	-636.24	1272.48	2.89	4	0.576
**Negative Affect**								
Not Imputed	Reduced	575.95	601.06	-280.97	561.95			
	Full	581.35	620.81	-279.68	559.35	2.59	4	0.628
Imputed	Reduced	558.57	583.70	-272.28	544.57			
	Full	564.67	604.17	-271.34	542.67	1.89	4	0.755

Note: ln-HF HRV = ln high frequency heart rate variability.

Trend-level Effects. Sample sizes include aggregated data from all participant assessments at each timepoint.

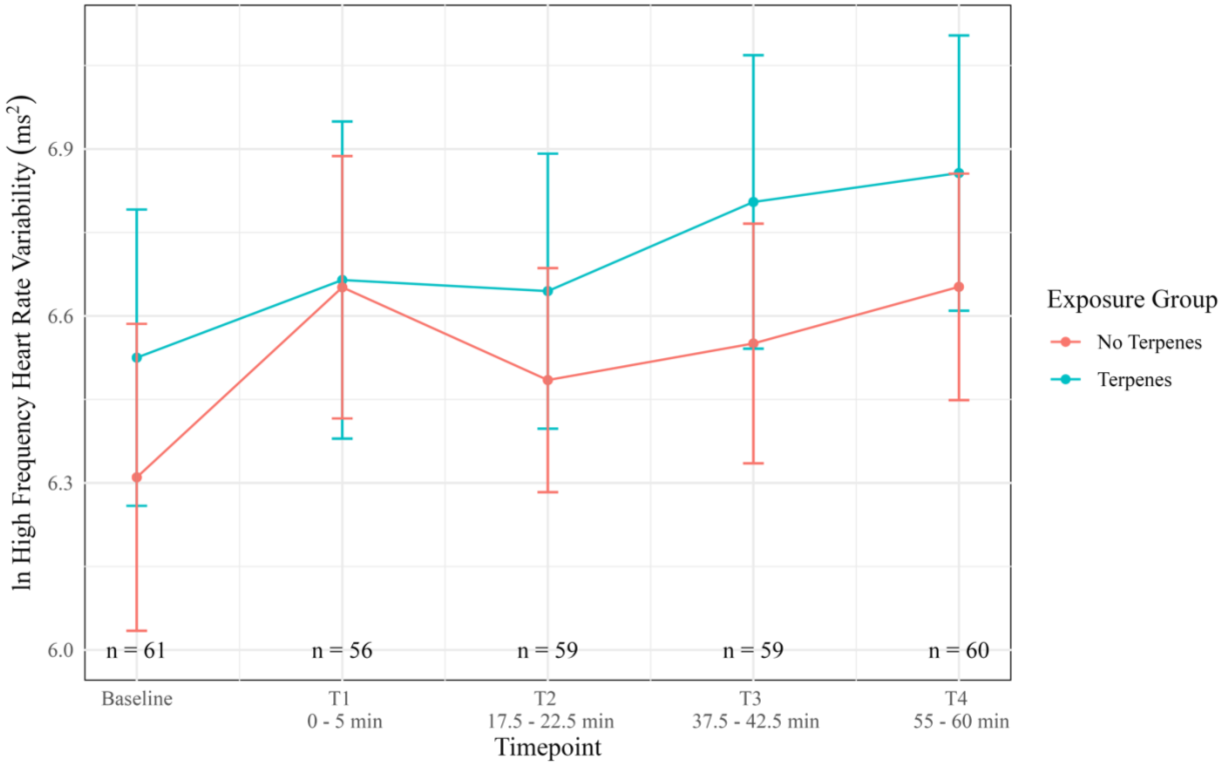

Figure 1. ln-HF HRV across time points comparing terpenes-on vs. terpenes-off filter conditions. Error bars represent the standard error of the mean for each filter condition.

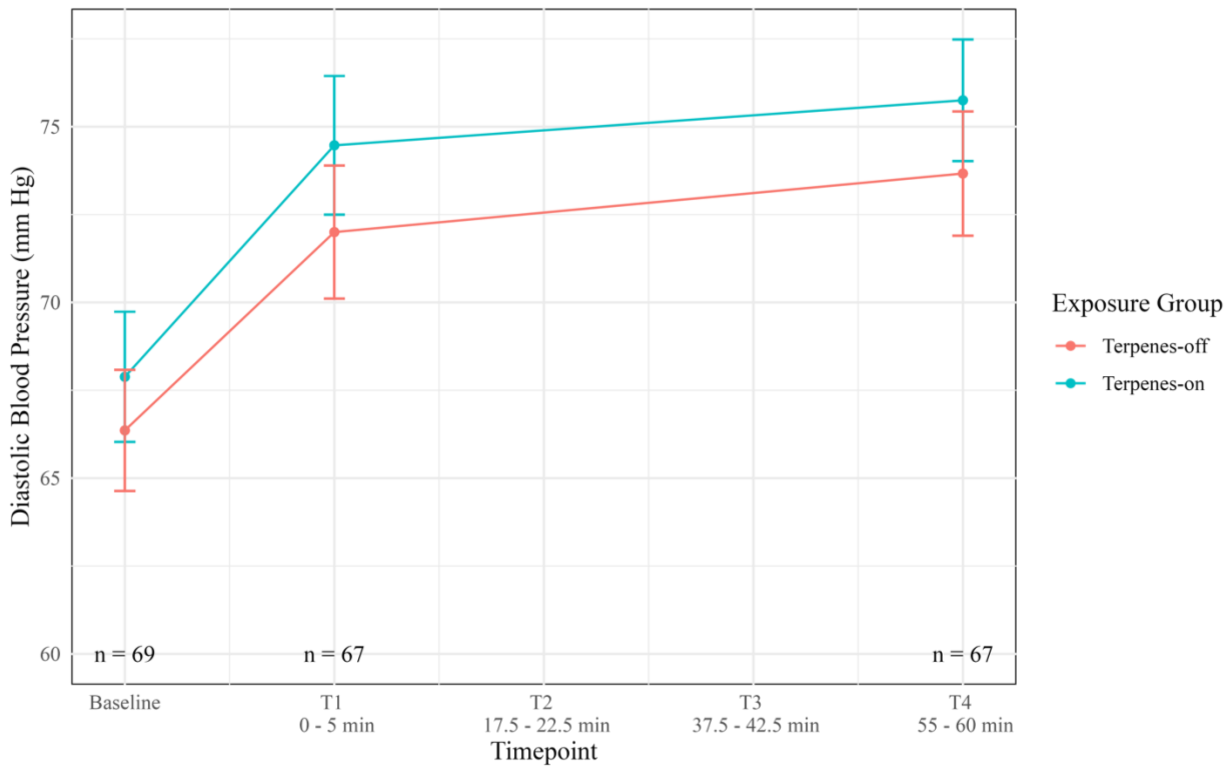

Figure 2. DBP across time points comparing terpenes-on vs. terpenes-off filter conditions. Error bars represent the standard error of the mean for each filter condition.

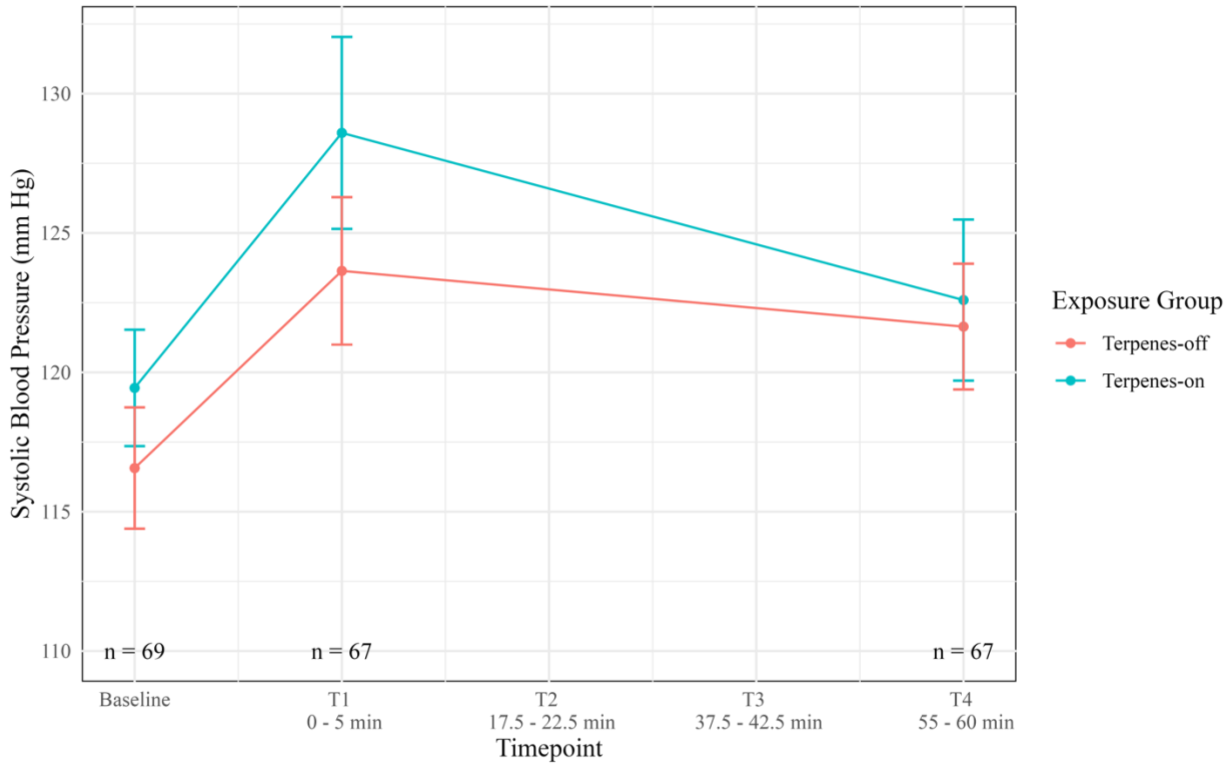

Figure 3. SBP across time points comparing terpenes-on vs. terpenes-off filter conditions. Error bars represent the standard error of the mean for each filter condition.

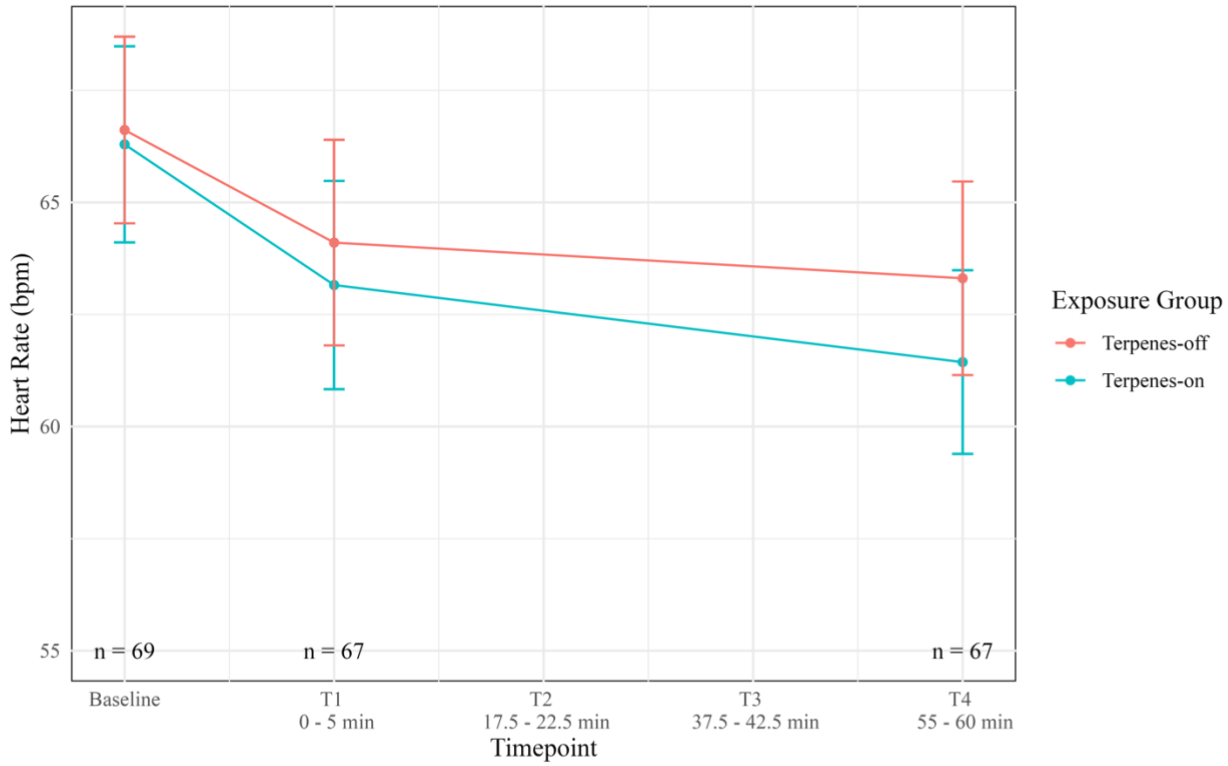

Figure 4. HR across time points comparing terpenes-on vs. terpenes-off filter conditions. Error bars represent the standard error of the mean for each filter condition.

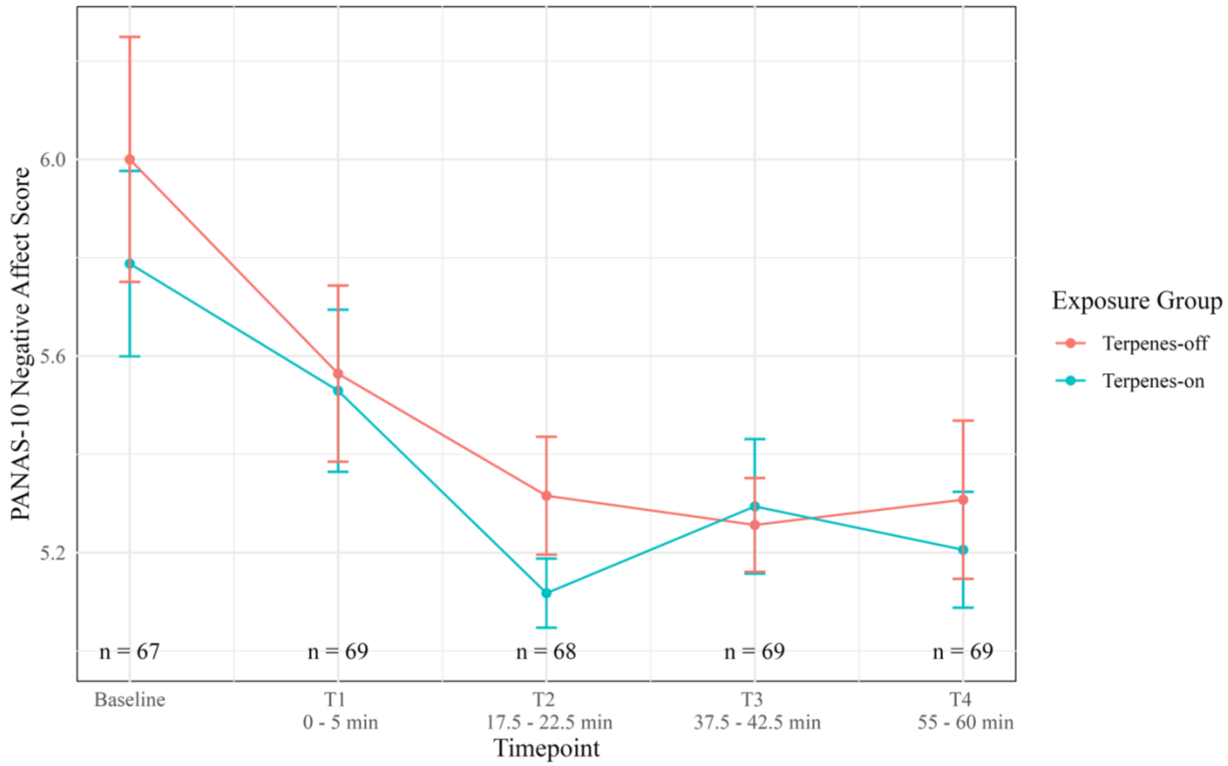

Figure 5. State negative affect across time points comparing terpenes-on vs. terpenes-off filter conditions. Error bars represent the standard error of the mean for each filter condition.

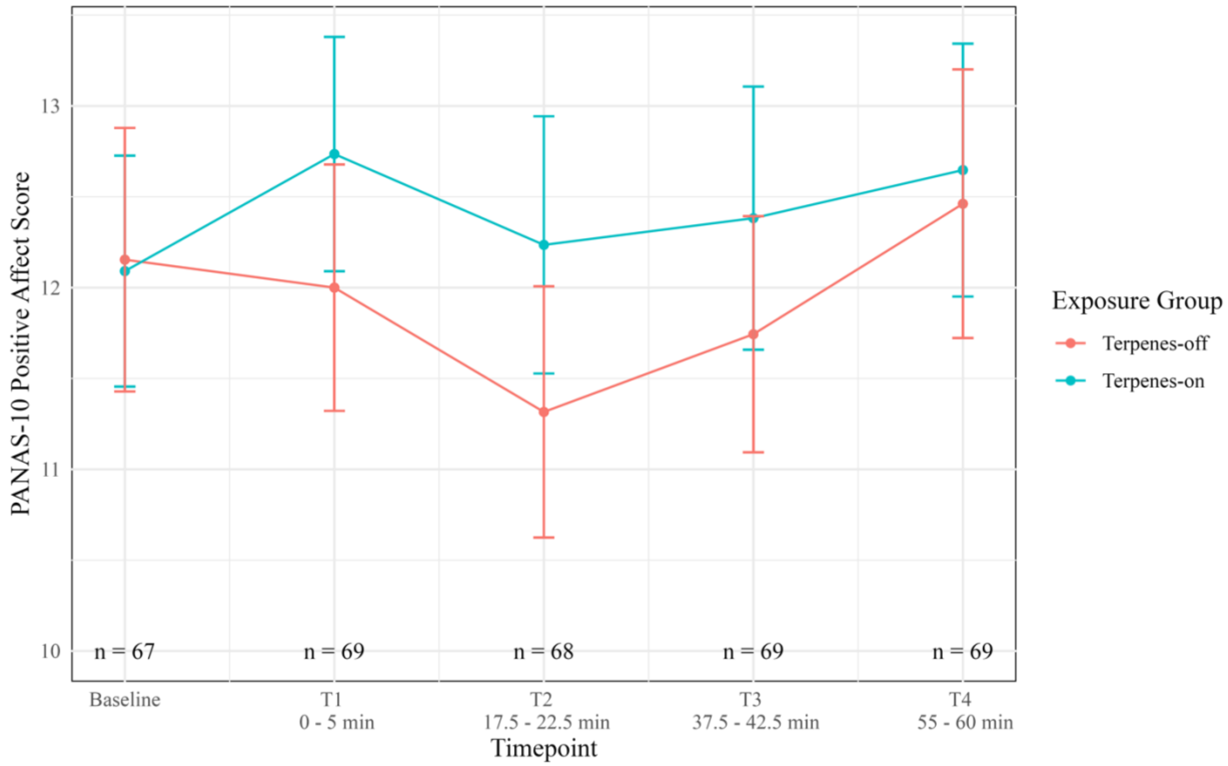

Figure 6. State positive affect across time points comparing terpenes-on vs. terpenes-off filter conditions. Error bars represent the standard error of the mean for each filter condition.

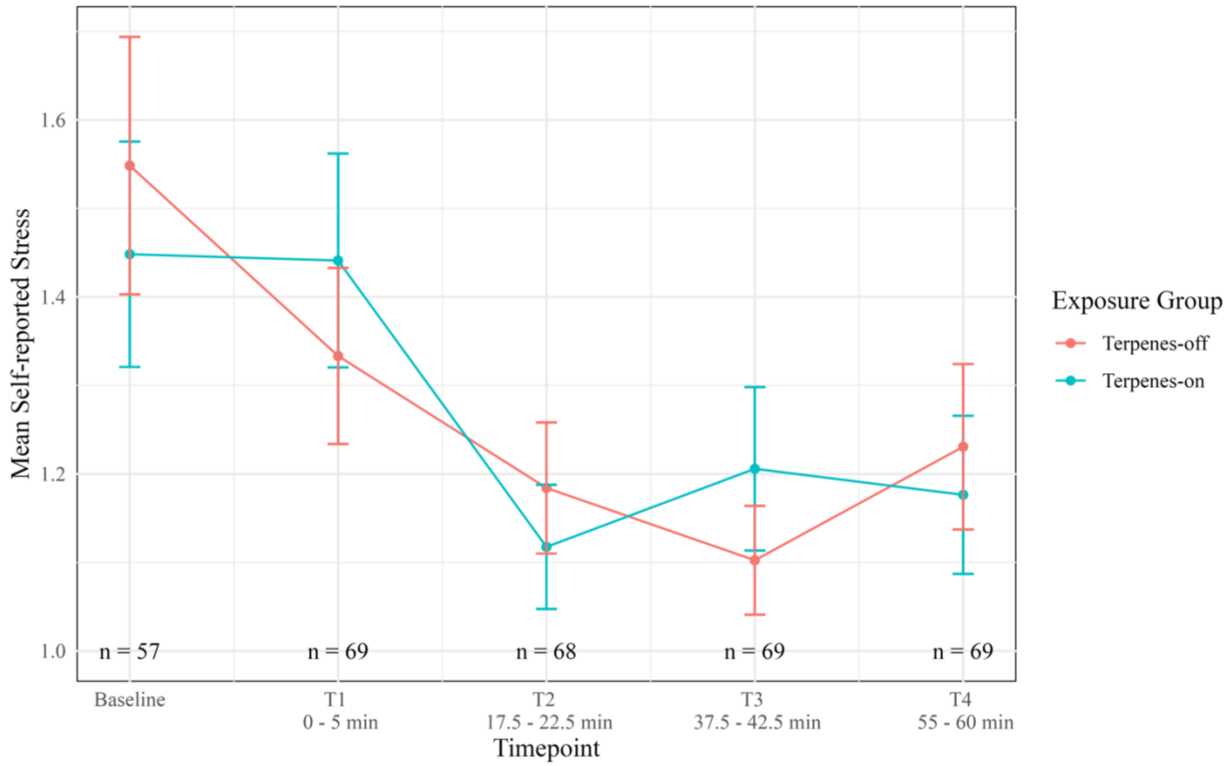

Figure 7. Self-reported state stress across time points comparing terpenes-on vs. terpenes-off filter conditions. Error bars represent the standard error of the mean for each filter condition.

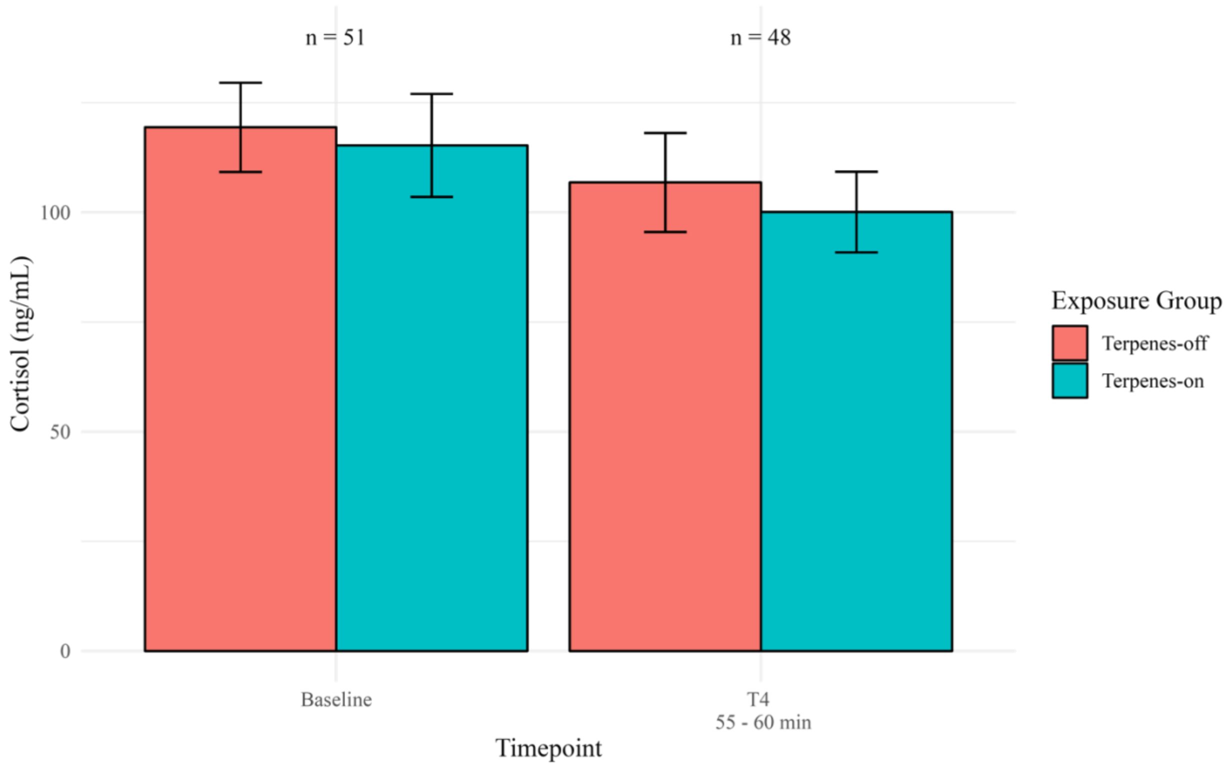

Figure 8. Cortisol across time points comparing terpenes-on vs. terpenes-off filter conditions. Error bars represent the standard error of the mean for each filter condition.

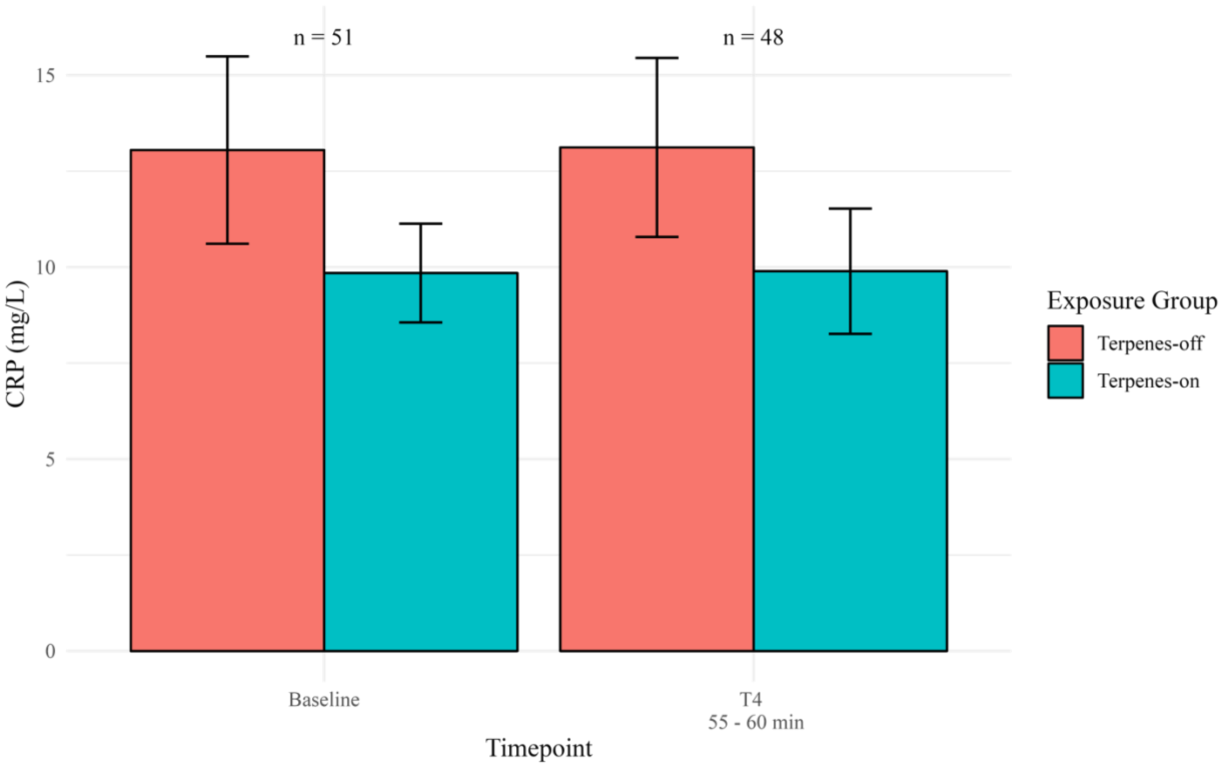

Figure 9. CRP across time points comparing terpenes-on vs. terpenes-off filter conditions. Error bars represent the standard error of the mean for each filter condition.

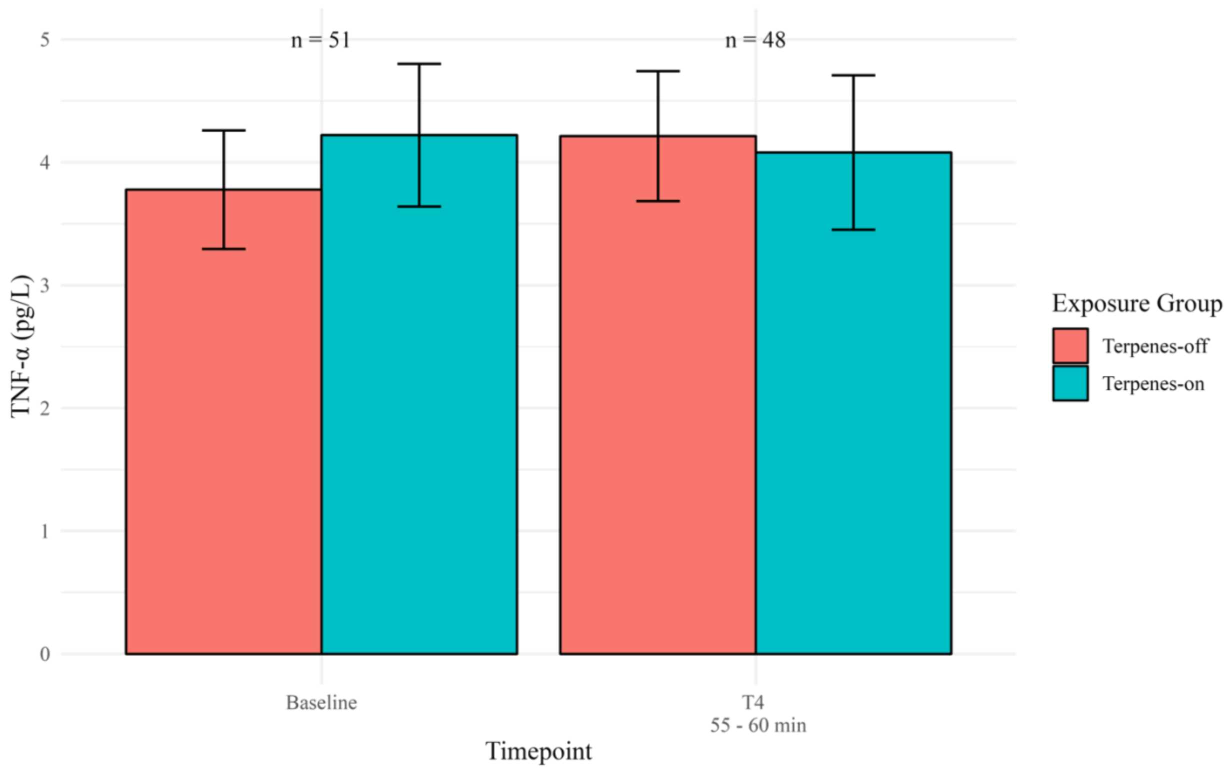

Figure 10. TNF-α across time points comparing terpenes-on vs. terpenes-off filter conditions. Error bars represent the standard error of the mean for each filter condition.

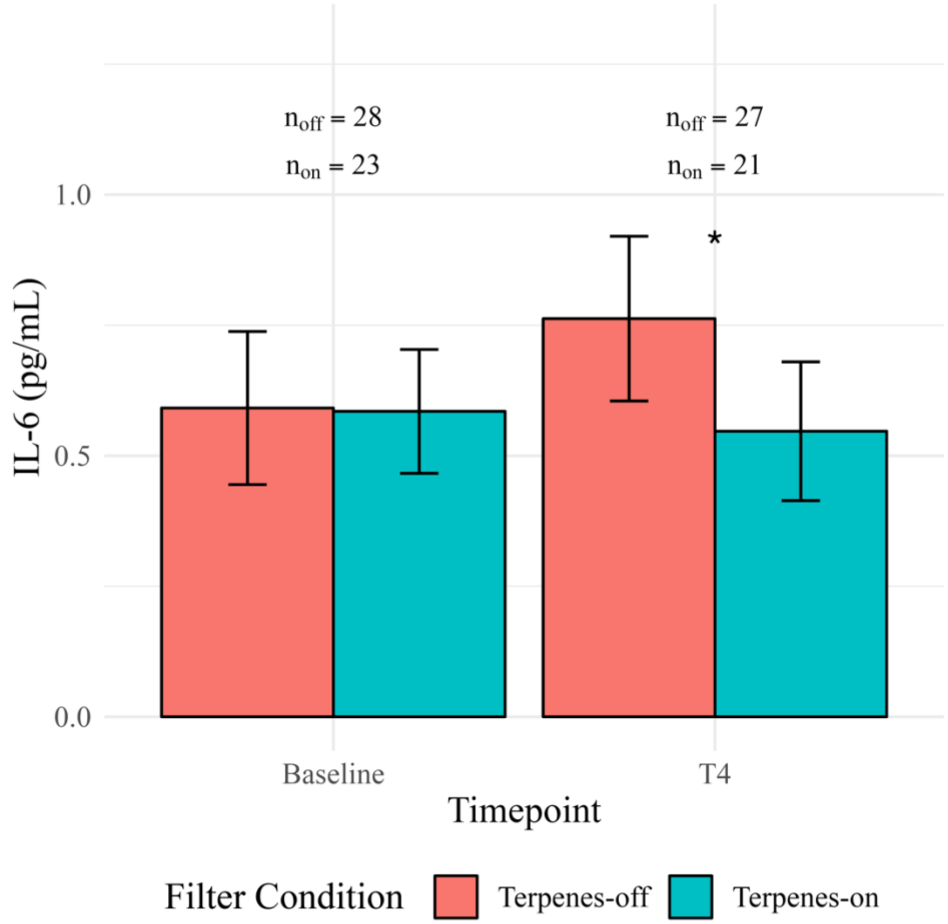

Figure 11. IL-6 across time points comparing terpenes-on vs. terpenes-off filter conditions. Error bars represent the standard error of the mean for each filter condition.

10. Association of Absorbed Dose with Outcomes

Sum composite absorbed dose was calculated as the sum of α-pinene, β-myrcene, Δ-3-carene, and limonene absorbed doses. Due to high (i.e., > 50%) proportions of non-detects, β-pinene and β-caryophyllene were excluded from the absorbed dose calculations.

Table 4. Associations of absorbed dose with study outcomes at T2 and T4.

	**α-Pinene**		**β-Myrcene**		**Δ-3-Carene**		**Limonene**		**Sum Composite**	
	Estimate (95% CI)	*p*	*Estimate* (95% CI)	*p*	*Estimate* (95% CI)	*p*	*Estimate* (95% CI)	*p*	*Estimate* (95% CI)	*p*
**ln-HF HRV**	1.69 (-9.27, 12.65)	0.765	0.83 (-2.82, 4.48)	0.660	5.54 (-4.87, 15.94)	0.306	0.65 (-0.99, 2.29)	0.442	1.06 (-0.38, 2.5)	0.161
**SCL**	-4.76 (-44.75, 35.23)	0.817	-4.90 (-22.01, 12.22)	0.579	-8.65 (-58.2, 40.89)	0.734	-3.54 (-11.93, 4.85)	0.413	-7.31 (-14.6, -0.03)	0.057
**DBP**	68.98 (-18.25, 156.21)	0.130	22.39 (-15.73, 60.5)	0.259	51.62 (-63.22, 166.47)	0.384	14.55 (-3.78, 32.88)	0.128	11.89 (-4.65, 28.42)	0.167
**SBP**	122.39 (-20.58, 265.37)	0.102	14.95 (-50.68, 80.58)	0.658	121.43 (-58.9, 301.76)	0.195	23.69 (-4.25, 51.62)	0.105	17.55 (-7.77, 42.88)	0.183
**HR**	35.28 (-34.06, 104.63)	0.326	7.41 (-24.08, 38.9)	0.648	63.70 (-21.78, 149.19)	0.153	-1.47 (-15.84, 12.9)	0.842	7.16 (-5.8, 20.11)	0.286
**PA**	1.04 (-38.08, 40.17)	0.959	12.65 (-4.11, 29.42)	0.149	30.86 (-17.7, 79.43)	0.221	1.50 (-6.69, 9.69)	0.722	3.75 (-3.6, 11.09)	0.324
**NA**	-1.80 (-8.5, 4.89)	0.601	-2.59 (-5.37, 0.18)	0.075	-2.40 (-10.94, 6.15)	0.586	0.79 (-0.52, 2.1)	0.246	0.15 (-1.11, 1.4)	0.821
**Stress**	-1.90 (-8.67, 4.86)	0.585	1.16 (-1.65, 3.98)	0.426	2.89 (-5.09, 10.87)	0.483	-0.61 (-1.92, 0.71)	0.375	-0.38 (-1.66, 0.89)	0.558
**Cortisol**	-535.04 (-973.19, -96.89)	**0.024**	116.86 (-85.47, 319.18)	0.272	-205.71 (-842.84, 431.42)	0.532	-67.13 (-197.17, 62.91)	0.321	-45.45 (-150.06, 59.17)	0.401
**IL-6**	-0.76 (-5.9, 4.37)	0.772	-1.67 (-3.90, 0.57)	0.153	-0.45 (-7.07, 6.18)	0.896	-0.22 (-1.68, 1.24)	0.769	-0.60 (-1.63, 0.43)	0.259
**TNF-α**	-10.65 (-36.5,	0.425	-10.63 (-22.07,	0.077	-21.42 (-55.40,	0.225	-3.01 (-10.16,	0.414	-5.73 (-10.95,	**0.038**

	**α-Pinene**		**β-Myrcene**		**Δ-3-Carene**		**Limonene**		**Sum Composite**	
	Estimate (95% CI)	*p*	*Estimate* (95% CI)	*p*	*Estimate* (95% CI)	*p*	*Estimate* (95% CI)	*p*	*Estimate* (95% CI)	*p*
	15.19)		0.82)		12.57)		4.13)		-0.51)	
**CRP**	-1.02 (-5.82, 3.77)	0.679	-1.00 (-3.17, 1.17)	0.371	-2.03 (-8.11, 4.06)	0.518	-0.48 (-1.76, 0.81)	0.473	-0.70 (-1.64, 0.25)	0.159

Note: ln-HF HRV = ln high frequency heart rate variability; SCL = skin conductance level; DBP = diastolic blood pressure; SBP = systolic blood pressure; HR = heart rate; PA = positive affect; NA = negative affect. Coefficients indicate the change in study outcomes associated with a 1 ug/L increase in absorbed dose.

In support of our hypothesis, we observed a statistically significant association between sum composite absorbed dose and TNF-α at time point 4. Specifically, a difference of 0.14 μg/L (IQR for sum composite absorbed dose) was associated with a difference in TNF-α of -0.79 pg/mL (95% CI: -1.51, -0.07; $p = 0.038$). With respect to individual terpenes, there was a statistically significant association between α-pinene absorbed dose and cortisol at time point 4, where a difference of 0.03 μg/L (IQR for α-pinene absorbed dose) was associated with a difference in cortisol of -16.05 ng/mL (95% CI: -29.20, -2.91; $p = 0.024$). No other associations between terpene absorbed doses and outcomes were significant. Given the lack of a consistent pattern of significant associations between absorbed dose and outcomes, as well as the small differences in levels of terpenes between filter conditions, these results are generally inconclusive.

8. Association of Absorbed Dose with the Pattern of Outcome Across Entire Duration of Session

In support of our hypothesis, we observed a statistically significant association between sum composite absorbed dose and ln-HF HRV ($p = 0.006$) and SCL ($p = 0.021$) response pattern over time. A difference of 0.14 µg/L of sum composite absorbed dose at time point 4 was associated with a difference in ln-HF HRV at time point 2 of 0.29 ms^2 (95% CI: 0.15, 0.44), 0.20 ms^2 at time point 3 (95% CI: 0.05, 0.35), and 0.14 ms^2 at time point 4 (95% CI: -0.01, 0.29). A difference of 0.14 µg/L of sum composite absorbed dose at time point 4 was associated with a difference in in SCL at time point 2 of -1.33 µS (95% CI: -2.38, -0.28), -1.59 µS at time point 3 (95% CI: -2.64, -0.54), and -1.47 µS at time point 4 (95% CI: -2.52, -0.43).

In support of our hypothesis, we observed a statistically significant association between Δ-3-carene absorbed dose and SCL response over time ($p = 0.034$). A difference of 0.02 µg/L (IQR for Δ-3-carene absorbed dose) of Δ-3-carene absorbed dose at time point 4 was associated with a difference in SCL of 0.10 µS at time point 2 (95% CI: -0.63, 0.84), -0.13 µS at time point 3 (95% CI: -0.86, 0.61), and -0.13 µS at time point 4 (95% CI: -0.87, 0.61). Counter to our hypothesis, we did not observe a significant association of any other terpene absorbed doses and study outcomes over time. As above, given the lack of a consistent pattern of significant associations between absorbed dose and outcomes, as well as the small differences in levels of terpenes between filter conditions, these results are generally inconclusive.

9. Association of Absorbed Dose and Study Outcomes ANOVA Results

Table 5. ANOVA results comparing full and reduced models to test the association of sum composite absorbed dose and the pattern of study outcome response.

Model	AIC	BIC	Log Likelihood	Deviance	Chisq	df	*p*
**ln-HF HRV**							
Reduced	147.71	167.94	-66.85	133.71			
Full	141.57	176.25	-58.78	117.57	16.14	5	0.006
**SCL**							
Reduced	841.10	862.80	-413.55	827.10			
Full	837.88	875.08	-406.94	813.88	13.22	5	0.021
**Positive Affect**							
Reduced	776.15	797.80	-381.07	762.1			
Full	779.43	816.56	-377.72	755.43	6.72	5	0.243
**Negative Affect**							
Reduced	307.79	329.45	-146.90	293.79			
Full	315.65	352.77	-145.82	291.65	2.15	5	0.829
**Stress**							
Reduced	124.31	144.65	-55.16	110.31			
Full	133.48	168.35	-54.74	109.48	0.83	5	0.975

Note: ln-HF HRV = ln high frequency heart rate variability; SCL = skin conductance level; DBP = diastolic blood pressure; SBP = systolic blood pressure; HR = heart rate; AIC = Akaike information criterion; BIC = Bayesian information criterion.

Table 6. ANOVA results comparing full and reduced models to test the association of α-pinene absorbed dose and the pattern of study outcome response.

	Model	AIC	BIC	Log Likelihood	Deviance	Chisq	df	*p*
**ln-HF HRV**								
	Reduced	147.71	167.94	-66.85	133.71			
	Full	148.41	183.10	-62.21	124.41	9.29	5	0.098
**SCL**								
	Reduced	841.10	862.80	-413.55	827.10			
	Full	846.64	883.83	-411.32	822.64	4.47	5	0.484
**Positive Affect**								
	Reduced	776.15	797.80	-381.07	776.15			
	Full	782.54	819.67	-379.27	758.54	3.61	5	0.607
**Negative Affect**								
	Reduced	307.79	329.45	-146.90	293.79			
	Full	315.47	352.59	-145.73	291.47	2.33	5	0.802
**Stress**								
	Reduced	124.31	144.65	-55.16	110.31			
	Full	131.52	166.38	-53.76	107.52	2.79	5	0.732

Table 7. ANOVA results comparing full and reduced models to test the association of β-myrcene absorbed dose and the pattern of study outcome response.

Model	AIC	BIC	Log Likelihood	Deviance	Chisq	df	*p*
**ln-HF HRV**							
Reduced	146.50	166.29	-66.25	132.50			
Full	148.01	181.95	-62.01	124.01	8.48	5	0.132
**SCL**							
Reduced	807.66	829.01	-396.83	793.66			
Full	812.83	849.43	-394.42	788.83	4.83	5	0.437
**Positive Affect**							
Reduced	724.89	746.20	-355.45	710.89			
Full	731.74	768.26	-353.87	707.74	3.16	5	0.676
**Negative Affect**							
Reduced	307.79	329.45	-146.90	293.79			
Full	313.06	350.19	-144.53	289.06	4.73	5	0.449
**Stress**							
Reduced	124.31	144.65	-55.16	110.31			
Full	126.80	161.66	-51.40	102.80	7.52	5	0.185

Note: ln-HF HRV = ln high frequency heart rate variability; SCL = skin conductance level; DBP = diastolic blood pressure; SBP = systolic blood pressure; HR = heart rate; AIC = Akaike information criterion; BIC = Bayesian information criterion.

Table 8. ANOVA results comparing full and reduced models to test the association of Δ-3-carene absorbed dose and the pattern of study outcome response.

Model	AIC	BIC	Log Likelihood	Deviance	Chisq	df	*p*
**ln-HF HRV**							
Reduced	147.71	167.94	-66.85	133.71			
Full	150.90	185.59	-63.45	126.90	6.80	5	0.236
**SCL**							
Reduced	841.10	862.80	-413.55	827.10			
Full	839.03	876.23	-407.52	815.03	12.07	5	0.034
**Positive Affect**							
Reduced	776.15	797.80	-381.07	762.15			
Full	782.40	819.52	-379.20	758.40	3.75	5	0.586
**Negative Affect**							
Reduced	307.79	329.45	-146.90	293.79			
Full	316.10	353.23	-146.05	292.10	1.69	5	0.890
**Stress**							
Reduced	124.31	144.65	-55.16	110.31			
Full	127.71	162.58	-51.86	103.71	6.60	5	0.252

Note: ln-HF HRV = ln high frequency heart rate variability; SCL = skin conductance level; DBP = diastolic blood pressure; SBP = systolic blood pressure; HR = heart rate; AIC = Akaike information criterion; BIC = Bayesian information criterion.

Table 9. ANOVA results comparing full and reduced models to test the association of limonene absorbed dose and the pattern of study outcome response.

Model	AIC	BIC	Log Likelihood	Deviance	Chisq	df	*p*
**ln-HF HRV**							
Reduced	147.71	167.94	-66.85	133.71			
Full	151.91	186.60	-63.96	127.91	5.80	5	0.327
**SCL**							
Reduced	841.10	862.80	-413.55	827.10			
Full	848.38	885.58	-412.19	824.38	2.72	5	0.743
**Positive Affect**							
Reduced	776.15	797.80	-381.07	762.15			
Full	781.20	818.32	-378.60	757.20	4.95	5	0.422
**Negative Affect**							
Reduced	307.79	329.45	-146.90	293.79			
Full	314.55	351.67	-145.27	290.55	3.25	5	0.662
**Stress**							
Reduced	124.31	144.65	-55.16	110.31			
Full	130.11	164.97	-53.05	106.11	4.20	5	0.521

Note: ln-HF HRV = ln high frequency heart rate variability; SCL = skin conductance level; DBP = diastolic blood pressure; SBP = systolic blood pressure; HR = heart rate; AIC = Akaike information criterion; BIC = Bayesian information criterion.

10. Smell Perception and Experience

Debrief interview responses and notes were coded to assess smell perception for each session. If a participant mentioned that they could not smell anything or that is was difficult to smell with

no description of their olfactory experience (i.e., pleasant or unpleasant), the session smell perception experience was coded as “Could not smell anything.” If a participant mentioned that they could smell the forest or other scents or that it was difficult to smell the forest but described their olfactory experience (i.e., pleasant or unpleasant), the session was coded as “Could smell the forest or other scents.” Finally, if the participant did not mention smells or the olfactory experience, the session was coded as “No mention of smell.” Additionally, participants were asked to rate the pleasantness of their sensory experience of the forest for sight, smell, and visual experience on a 10-point scale ranging from 1 (“not pleasant”) to 10 (“extremely pleasant”). Summary statistics are presented in Table 10.

Table 10. Sensory perception and pleasantness stratified by condition.

	**Terpenes-on filter** (N=32 sessions)	**Terpenes-off filter** (N=37 sessions)	**Overall** (N=69 sessions)
**Smell Perception**			
Could not smell anything	12 (37.5%)	14 (37.8%)	26 (37.7%)
Could smell the forest or other scents	6 (18.8%)	2 (5.4%)	8 (11.6%)
No mention of smell	14 (43.8%)	20 (54.1%)	34 (49.3%)
Missing	0 (0%)	1 (2.7%)	1 (1.4%)
**Smell Pleasantness**			
Mean (SD)	6.45 (2.23)	6.83 (2.18)	6.65 (2.19)
Median [Min, Max]	7.00 [2.00, 10.0]	6.00 [3.00, 10.0]	6.00 [2.00, 10.0]
Missing	1 (3.1%)	2 (5.4%)	3 (4.3%)
**Visual Pleasantness**			
Mean (SD)	9.09 (1.03)	9.03 (1.21)	9.06 (1.12)
Median [Min, Max]	9.00 [6.00, 10.0]	9.00 [5.00, 10.0]	9.00 [5.00, 10.0]
Missing	0 (0%)	1 (2.7%)	1 (1.4%)
**Sound Pleasantness**			
Mean (SD)	7.61 (2.01)	7.63 (2.18)	7.62 (2.09)
Median [Min, Max]	8.00 [2.00, 10.0]	7.00 [4.00, 10.0]	8.00 [2.00, 10.0]
Missing	1 (3.1%)	2 (5.4%)	3 (4.3%)

18.8% of participants in the terpenes-on filter condition mentioned being able to smell the forest or other scents. 5.4% of participants in the terpenes-off filter condition mentioned being able to smell the forest or other scents (see Figure 11).

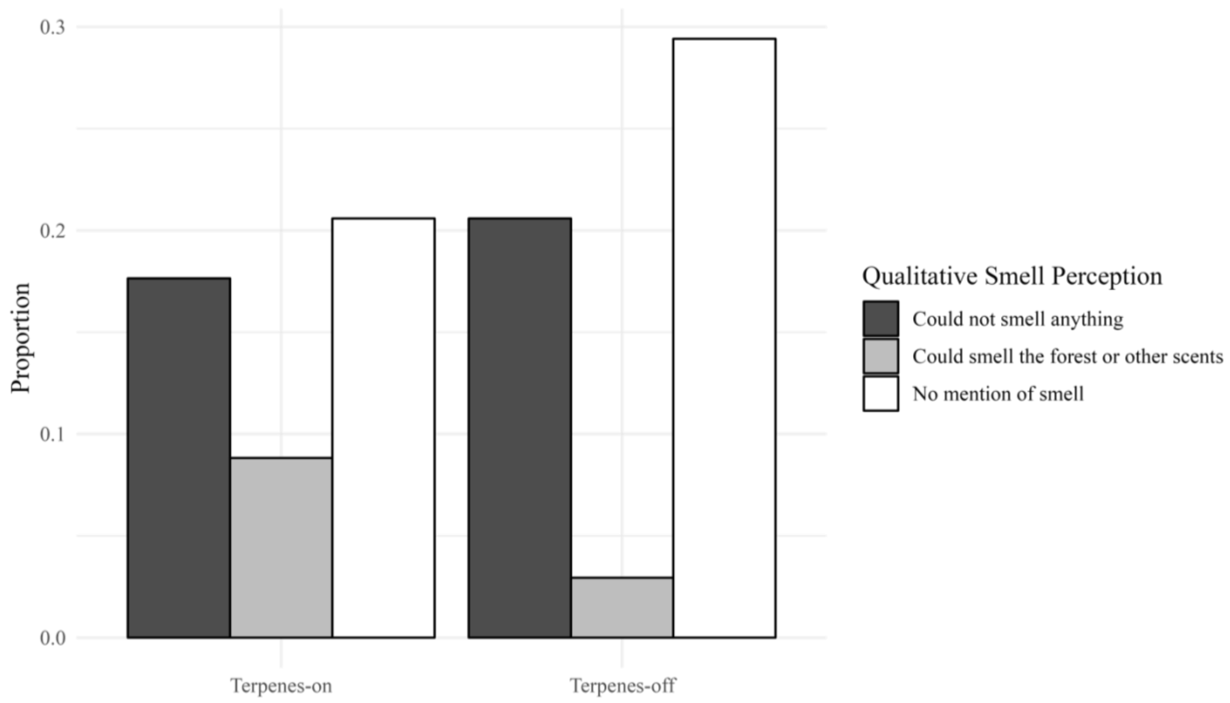

Figure 11. Participant smell perception by filter condition.

Mean smell pleasantness ratings for each exposure were similar (6.45± 2.23 for terpenes-on filter condition and 6.83±2.18 for terpenes-off filter condition). Only 16.6% of participants were able to correctly identify when they had been exposed to the terpenes-on filter condition, while 76.6% could not identify a difference in smell between conditions and 6.6% indicated that they thought the terpenes-off filter condition had the more pleasant olfactory experience (see Figure 12).

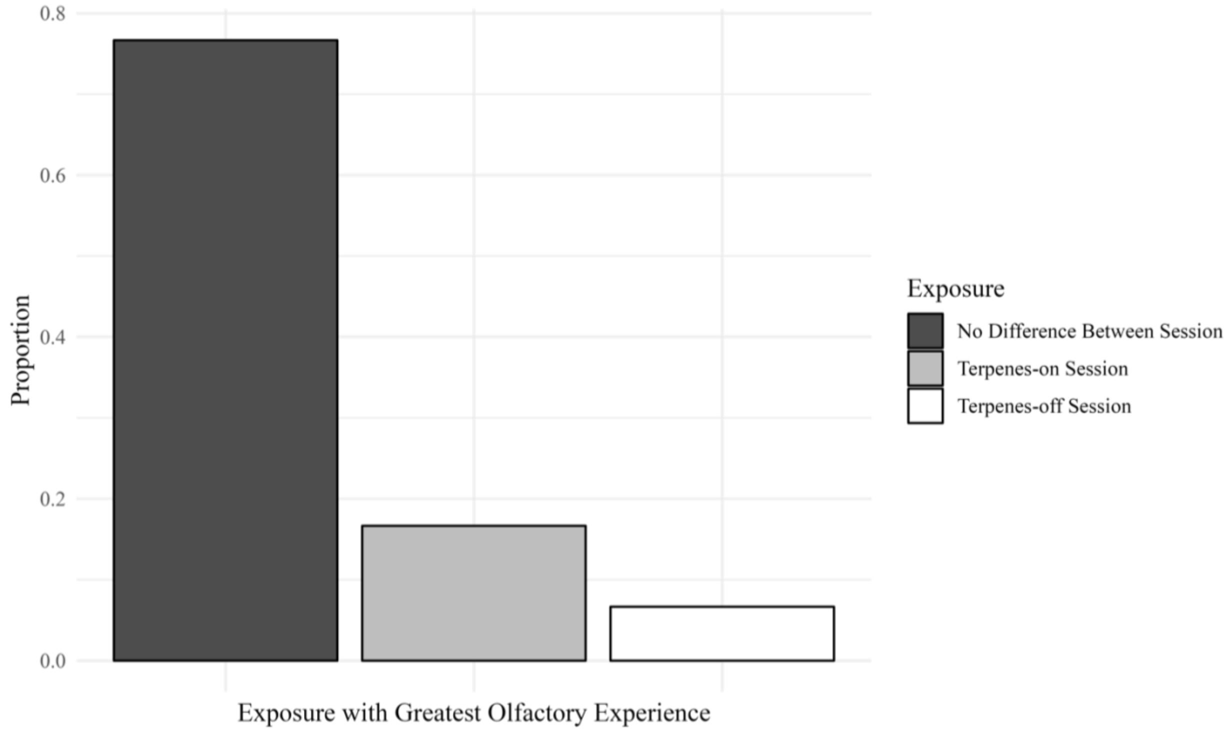

Figure 12. Participant report of condition with the olfactory experience that was most detectable.
